# Genetic screens reveal mechanisms for the transcriptional regulation of tissue-specific genes in normal cells and tumors

**DOI:** 10.1093/nar/gkz080

**Published:** 2019-02-07

**Authors:** Ikrame Naciri, Marthe Laisné, Laure Ferry, Morgane Bourmaud, Nikhil Gupta, Selene Di Carlo, Anda Huna, Nadine Martin, Lucie Peduto, David Bernard, Olivier Kirsh, Pierre-Antoine Defossez

**Affiliations:** 1 Univ. Paris Diderot, Sorbonne Paris Cité, Epigenetics and Cell Fate, UMR 7216 CNRS, 75013 Paris, France; 2 INSERM U1132 and USPC Paris-Diderot, Hôpital Lariboisière, Paris, France; 3 Unité Stroma, Inflammation & Tissue Repair, Institut Pasteur, 75724 Paris, France; INSERM U1224, 75724 Paris, France; 4 Centre de Recherche en Cancérologie de Lyon, Inserm U1052, CNRS UMR 5286, Université de Lyon, Centre Léon Bérard, 69008 Lyon, France

## Abstract

The proper tissue-specific regulation of gene expression is essential for development and homeostasis in metazoans. However, the illegitimate expression of normally tissue-restricted genes—like testis- or placenta-specific genes—is frequently observed in tumors; this promotes transformation, but also allows immunotherapy. Two important questions are: how is the expression of these genes controlled in healthy cells? And how is this altered in cancer? To address these questions, we used an unbiased approach to test the ability of 350 distinct genetic or epigenetic perturbations to induce the illegitimate expression of over 40 tissue-restricted genes in primary human cells. We find that almost all of these genes are remarkably resistant to reactivation by a single alteration in signaling pathways or chromatin regulation. However, a few genes differ and are more readily activated; one is the placenta-expressed gene ADAM12, which promotes invasion. Using cellular systems, an animal model, and bioinformatics, we find that a non-canonical but druggable TGF-β/KAT2A/TAK1 axis controls ADAM12 induction in normal and cancer cells. More broadly, our data show that illegitimate gene expression in cancer is an heterogeneous phenomenon, with a few genes activatable by simple events, and most genes likely requiring a combination of events to become reactivated.

## INTRODUCTION

The human body contains ∼200 cell types, each characterized by a specific gene expression pattern. This pattern itself is determined by transcription factors, acting on a chromatin template rendered more or less permissive to their action by chromatin-modifying factors, such as DNA methyltransferases and demethylases, histone modifying enzymes, and nucleosome remodelers (1,2). These gene expression events are also influenced by cellular signaling pathways, which transmit the intracellular and extracellular signals that the cell is subjected to during development and during its normal life (3,4). A well-known example of extracellular signal is the cytokine Transforming Growth Factor β (TGF-β), which plays complex roles during development, immunity and cancer (5). Transcriptional regulation by chromatin-templated processes and cellular signaling have each been studied extensively individually, yet the interplay between these two processes has been harder to decipher. A few examples of kinase signaling cascades influencing chromatin status have been reported (6,7), but these findings have not been generalized.

Cancer cells show abnormalities in signaling and in chromatin regulation, leading to illegitimate gene expression, i.e. the expression of a gene in a tissue type where it is normally silenced (8). This illegitimate expression can contribute to tumorigenesis (9), however the inappropriate expression of tissue-specific genes in tumors gives a sensitive and robust diagnostic tool (10). In addition, the mis-expressed genes may produce immunogenic proteins, and render the tumor cells amenable to immunotherapy (11,12). Many of the tissue-restricted genes that are illegitimately re-expressed in tumor cells are normally only expressed in the testis; these genes are called Cancer/Testis (C/T) genes (13). However, other tissue-restricted genes, and in particular placental genes, may also be reactivated in tumors (10).

The goal of the present work was to identify chromatin regulators and signaling kinases which could be involved in illegitimate gene expression, to determine the interconnection between these molecular actors, and to test the physiological relevance of these findings.

Using high-throughput unbiased approaches, we report that most tissue-restricted genes examined are remarkably resistant to reactivation by a single hit in signaling pathways or chromatin regulators, suggesting that their reactivation in cancer results from a combination of events occurring during transformation.

An exception to this rule is the developmental gene ADAM12, highly expressed in the placenta, which encodes a metalloprotease re-expressed in cancers of diverse origins, such as breast, lung, liver, and colon malignancies (14–18). The oncogenic role of ADAM12 is especially clear in the case of Triple-Negative Breast Cancer (19).

We find that ADAM12 can be robustly induced in normal lung cells by stimulating MAP3K7/TAK, a kinase in the non-canonical TGF-β signaling pathway (20). This provides a mechanism for the known responsiveness of ADAM12 to TGF-β in cancer cells (21–25). ADAM12 can also be induced by depleting the histone deacetylase SIRT6 or the histone acetyltransferase GCN5/KAT2A. This repressive role of KAT2A is unusual, and we explain it by showing that KAT2A acts upstream of TAK1 and interacts with TAK1. Finally, our bioinformatic analyses argue that these mechanisms are physiologically relevant in the context of human cancer.

These data show that TAK1 inhibition by existing, well-tolerated drugs, could be an avenue to prevent illegitimate ADAM12 induction and decrease transformed phenotypes in several cancer types. More broadly, they describe unexpected connections between signaling pathways and chromatin regulators, and they reveal rules underpinning tissue-specific gene regulation in normal cells and tumors.

## MATERIALS AND METHODS

### Reagents and antibodies

The following antibodies were employed in this study: mouse ADAM12 (Proteintech 14139-1-AP); human ADAM12 (Sigma HPA030867); human TAK1 (SCBT sc-1839); human KAT2A (SCBT sc-20698); human SIRT6 (Abcam ab62739); human SMAD3 (ab28379), human phospho-SMAD3 (Abcam ab52903), human tubulin (Abcam ab7291), human TAB1 (CST 3226); human Histone H3 (CST 2650). TGF-β was from Proteintech and the TAK1 inhibitor (5Z)-7-oxozeaenol from Sigma.

### Cell culture

MRC5, IMR90, SW39, SUM159PT, MDA-MB-231 and HEK293T were cultured in DMEM medium supplemented with 10% FBS and 1% penicillin/Streptomycin. BT549 cells were cultured in RPMI 1640 medium supplemented with 10% FBS and 1% penicillin/streptomycin. All the cell lines were cultured in a humidified atmosphere at 37°C under 5% CO_2_. The identity of all the cell lines was verified using the Eurofins cell line authentication service. We are grateful to Annabelle Decottignies and Woodring Wright for the gift of SW39 cells.

### Virus production

The production of retroviruses and infection by the library was done as described in our previous publication (26). The plasmids expressing myristoylated kinases were a gift from William Hahn and Jean Zhao (Addgene kit # 1000000012; list of the kinases in Supplementary Table S1). All the plasmids were grown and prepared individually; twenty randomly selected vectors were sequenced and all contained the expected insert. IMR90 were seeded into six-well plates, infected, and total RNA was recovered 4 days after infection.

### siRNA screening

For the siRNA screen seventy thousand IMR90 cells were seeded per well in six-well plates, reverse transfected with siRNAs using Dharmafect 1 (Dharmacon, Horizon Discovery), and total RNA was extracted 5 days after transfection. The protocol is described in detail elsewhere (27). For the screen we used ON-TARGETplus siRNA SMARTpools (Dharmacon, GE Healthcare), as well as control non-targeting pools (list of genes targeted in Supplementary Table S4). Additional siRNAs against TAK1, SIRT6 and KAT2A were purchased from Dharmacon and Sigma (sequences in Supplementary Table S6).

### Selection of the genes of interest

We included 42 genes in the expression analysis (in addition to normalizers and other controls). These 42 genes were chosen according to several criteria. We first selected 20 tissue-specific genes inappropriately re-expressed in cancer from the data of Rousseaux and colleagues (10). We chose ten genes for which re-expression correlates with loss of DNA methylation as judged by 450K array data (MAGEB6, BRDT, DPEP3, RNF17, DDX4, SPATA22, TPTE, TUBA3C, DAZL, C10orf82), and 10 genes for which there is no such correlation (ADAM2, ADAM12, ADCY10, ASZ1, C9orf11, ALAS1, DDX53, ATAD2, RFX4, HORMAD1). We selected five 5-aza-cytidine inducible cancer/testis genes (MAGEA3, MAGEA4, MAGEA12, NY-ESO-1/CTAG1B and TKTL1) from a different publication (28). The rest of the genes were chosen from literature searches.

### Nanostring analysis

The analysis was done by the Genomics Platform of Institut Curie (Paris, France). RNAs were analyzed with the BioAnalyzer using a Nano LabChip to assess their integrity (Bioanalyzer 2100 RNA 6000 Nano Kit From Agilent Technologies) and with a Nanodrop (Thermo) to assess their purity and concentration. RNA abundance was then measured with Nanostring technology (Nanostring Flex nCounter analysis system). All RNA processed to analysis displayed a RIN >7.6 and a 260 nm/280 mm Ratio >1.8. Raw counts were first normalized with internal controls (Nanostring POS controls) and with the expression of three housekeeping genes (PGK1, TBP and TUBB2A). Nanostring probes are listed in Supplementary Table S2. The probes were tested against ‘Human Reference Total RNA’ (Agilent #750500), a mixture of mRNA from 10 human cancer cell lines (breast adenocarcinoma, cervix adenocarcinoma, hepatoblastoma, glioblastoma, melanoma, liposarcoma, histiocytic lymphoma, T lymphoblastic leukemia, plasmacytoma, and testicular embryonal carcinoma).

### Statistical analysis of Nanostring data

For each probe, we plotted the distribution of log2 normalized counts for all samples (controls and experiments). In the figures, we indicate two thresholds: mean + 2.5 standard-deviations, and mean + 3.5 standard-deviation. For normal distributions, these values correspond to a *P*-value of 1% and 0.05% after a two-tailed *t*-test, respectively. A Shapiro test showed that not all distributions were normal, so we did not indicate *P*-values in the figures.

### Quantitative real-time PCR

RNA extraction was doing using Tri reagent according to the manufacturer's recommendations. RNA was DNase treated, reverse transcribed using Superscript III (Invitrogen) and Oligo dT primers. qPCR was performed using Power SYBR Green (Applied Biosystems) on a ViiA 7 Real-Time PCR System (LifeTech), as described previously (29). TBP and PGK1 genes were used for normalization of expression values. The sequence of qRT-PCR is given in Supplementary Table S5.

### Immunobloting

Cells were harvested and lysed in RIPA buffer with protease and phosphatase inhibitors, sonicated (series of 30 s ON, 30 s OFF during for 5–10 min; Bioruptor, Diagenode). Protein extract was reduced with NuPage sample reducing agent and LDS sample buffer (LifeTech) as previously described (30). Fifty micrograms of protein was loaded for each sample.

### Concanavalin enrichment (ADAM12 western blots)

Cells were harvested and lysed in RIPA buffer with protease and phosphatase inhibitors, sonicated (series of 30 s ON, 30 s OFF during for 5–10 min; Bioruptor, Diagenode). Protein extract from 200 and 400 μg of protein was incubated overnight with concanavalin A beads. The beads were the washed five times with RIPA buffer and LDS sample buffer and sample reducing agent was had added to the beads. The beads were boiled during for 5 min at 95°C and resolved by SDS–polyacrylamide gel electrophoresis. Proteins separated by electrophoresis were and electroblotted onto a nitrocellulose membrane using standard protocols (31).

### Treatment of mice with (5Z)-7-oxozeaenol

M12CIG mice (32) were injected intraperitoneally with TAK1 inhibitor (5 mg/kg) one day prior to cardiotoxin injury and every day subsequent to injury. Cardiotoxin injury was performed as follows: anesthetized mice were injected with 50 μl of 10 μM cardiotoxin (Latoxan) in tibialis anterior muscles. Three days after injury the mice were sacrificed and the muscles were dissected, with half of each muscle used for protein extraction and the other half fixed with paraformaldehyde for immunofluorescence. The sections were stained with anti-GFP antibodies (Thermo Fisher #A11122, 1:1000) and counterstained with Alexa Fluor 488 anti-rabbit antibody (Invitrogen).

### Co-Immunoprecipitation

Total protein extract was prepared by mixing cells with lysis buffer as previously described (33,34). The extract was incubated overnight in a cold room with agitation in the presence of 2 μg TAK1 antibody pre-incubated with magnetics beads coupled to protein G (Invitrogen) for 2 h. The beads were then washed seven times with 1 ml of wash buffer. The adsorbed proteins were dissociated by boiling beads for 10 min in 24 μl of Laemmli buffer and resolved by SDS-polyacrylamide gel electrophoresis. Proteins were separated by electrophoresis and electroblotted onto a nitrocellulose filter as previously described (35).

### Wound healing assay

SUM159PT cells were plated in 12-well dishes and grown to confluence. During this time, cells were treated with TAK1 inhibitor or vehicle (DMSO). Then a scratch was performed in the middle of the well using a P10 plastic tip and image acquisition was performed every two hours using the Incucyte system.

### TCGA analysis

TCGA gene count datasets for lung, colon and breast normal and cancer samples were downloaded from *Recount2* (36). ADAM12 expression normalized with *DESeq2* (37) was used to stratify tumors. We selected subgroups of tumors belonging to the first decile and the last decile of ADAM12 expression, and we compared the expression of TAK1, KAT2A and SIRT6 in these groups relative to the respective healthy tissue.

### TAK1 signature characterization, TAK1 activation score, and correlation analysis

The TAK1 signature gene set was defined using public microarray data (GSE65069), re-analyzed with the *LIMMA* package (38). Using this data set, we first identified 516 genes that respond to TGF-β with a Fold-Change >1.5 and a *P*-value <0.05. Then we identified the 190 genes that lost induction by TGF-β in the presence of the TAK1 inhibitor (5Z)-7-oxozeaenol. This list is called the ‘TAK1 signature’ and is presented in Supplementary Table S7.

For correlations, we compute a ‘TAK1-activation score’, as the sum of the fold changes of the 190 genes in the TAK1 signature, in a particular tumor relative to normal tissue.

Lastly, we computed the Pearson correlation between this TAK-activation score and *ADAM12* or *KAT2A* expression. To test the significance of this correlation, we performed 1000 drawings of 190 random genes, and calculated the Pearson correlation between each random signature and the expression of ADAM12 or KAT2A. The distribution of correlation values was plotted, and the *P*-value of the correlation for the TAK1 signature was estimated using the Gumbel approximation.

## RESULTS

### Most tissue-restricted genes are not illegitimately induced by a single hit in signaling pathways

**Figure 1.**
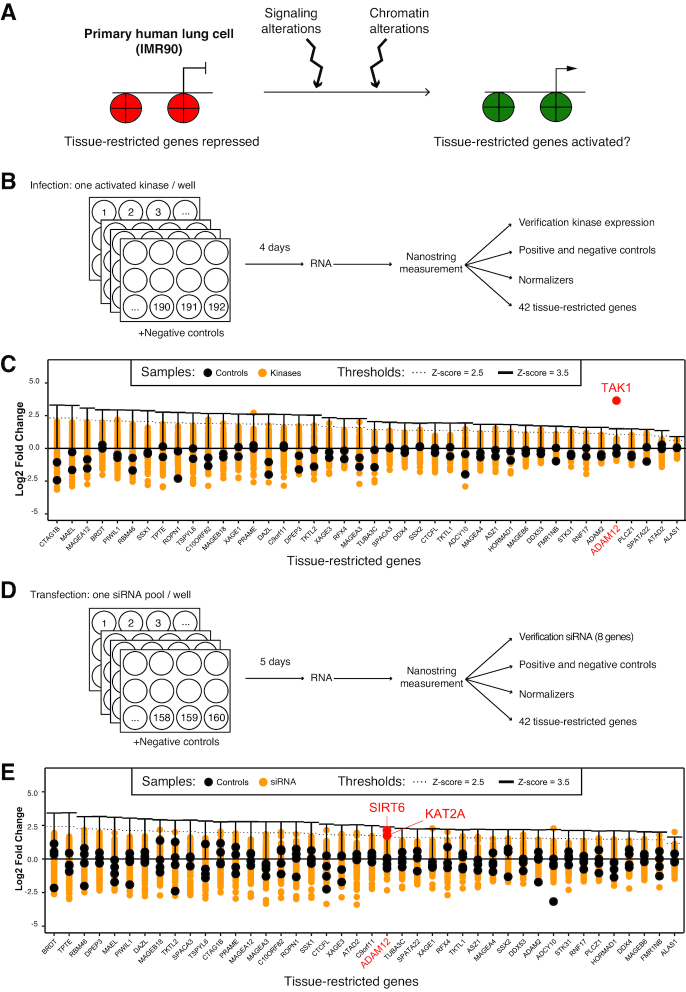
Two screens to investigate the mechanisms controlling tissue-restricted gene reactivation converge on ADAM12. (**A**) Principle of the approach. Primary human cells were challenged by alterations in signaling pathways or chromatin regulators, and we determined whether these changes were sufficient to cause inappropriate expression of tissue-restricted genes. Red is used to represent repressive chromatin, and green permissive chromatin. (**B**) Design of the signaling screen: the primary cells were infected with 192 different genetically activated kinases, and gene expression assayed by Nanostring. (**C**) Results of the signaling screen: log_2_ fold change of expression for each of the 42 tissue-restricted genes following infection by the 192 activated kinases. Each infection is represented by a yellow dot. The negative controls are indicated as black dots. The only kinase/gene pair showing a *Z*-score >3.5 is the gene ADAM12 being highly activated by the kinase MAP3K7/TAK1 (red dot). (**D**) Design of the chromatin screen: the primary cells were transfected with 160 different siRNA pools, each targeting a specific chromatin regulator, then gene expression was assayed by Nanostring. (**E**) Results of the chromatin screen: log_2_ fold change of expression for each of the 42 tissue-restricted genes following transfection of the 160 siRNA pools. Each transfected sample is shown as a yellow dot, controls are shown as black dots. Only a few siRNA/gene pairs have a *Z*-score >2.5. These include the induction of ADAM12 by depletion of SIRT6 and KAT2A.

We set out to determine whether alteration in signaling pathways, or alteration of chromatin factors, could reverse the silencing of tissue-restricted genes in otherwise normal cells. For this we developed two genetic screens, using as a model the non-transformed human lung cells IMR90 (Figure 1A).

We first sought to identify signaling pathways that, when activated, could turn on tissue-restricted gene expression (Figure 1B). For this, we used a collection of 192 kinases (list in Supplementary Table S1), genetically activated by the addition of a myristoylation signal, and encoded by retroviral vectors (39). IMR90 cells were grown in wells, and each well was infected with an individual kinase; in addition some wells were infected with the matching empty retroviral vector (control). After infection we extracted total RNA and used Nanostring probes to measure: the expression of the activated kinases (using the common sequence encoding the myristoylation-Flag tag); the abundance of spike-in positive and negative controls; the expression of four housekeeping genes for normalization; and the expression of a diverse set of 42 tissue-restricted genes (list of probes in Supplementary Table S2). These genes are expressed weakly or not at all in IMR90, and were chosen to reflect the distribution of illegitimately expressed genes in cancer, with a majority of testis-specific genes, some placenta-specific genes, and a few other cases (list and characteristic of genes in Supplementary Table S3, criteria for selection of the genes explained in the material and methods sections). The 42 genes under study were also chosen to represent potentially different mechanisms of epigenetic silencing. For instance, 23 of the genes contain a CpG island, and of those 18 are partially or fully methylated in IMR90 (Supplementary Table S3). The 19 genes that do not contain a CpG island are likely repressed by a DNA methylation-independent mechanism.

We included in the sample series, as positive controls, RNA from transformed IMR90 derivatives (SW39 cells), and a mixed RNA sample prepared from 10 different human cancer cell lines (Breast adenocarcinoma; cervix adenocarcinoma; hepatoblastoma; glioblastoma; melanoma; liposarcoma; histiocytic lymphoma; T lymphoblastic leukemia; plasmacytoma; and testicular embryonal carcinoma). We observed that 38 of the 42 tissue-restricted gene probes showed 2-fold or more increased signal relative to control in SW39 or in mixed human cancer mRNA, showing their potential to detect an increased signal (Supplementary Figure S1A).

We also measured the expression of the myristoylated kinases (using the common sequence encoding the myristoylation-Flag tag); all were detected, whereas non-infected cells IMR90 or SW39) showed no expression of the tag (Supplementary Figure S1B).

Having thus validated the expression of the kinases, and the ability of the Nanostring probes to detect an induction in gene expression, we next analyzed the expression of the 42 tissue-restricted genes in the ∼200 samples (control and infected cells). The normalized data are presented in Figure 1C. They show, strikingly, that very few of the ∼8400 probe/kinase pairs tested show a *Z*-score >2.5 (this corresponds to *P* = 1% assuming the data distribution is normal). One notable outlier is the gene ADAM12, which was strongly induced by the kinase MAP3K7/TAK1 (*Z*-score = 8.47, *P*-value < 10^−5^).

### Most tissue-restricted genes are not illegitimately induced after depletion of a single chromatin factor

The second genetic screen we carried out aimed at determining whether the depletion of chromatin regulators (by RNAi) could be sufficient to reactivate tissue-restricted genes in otherwise normal cells. A further goal of this screen was to determine whether the genes reactivated by signaling pathway alterations were also sensitive to the depletion of chromatin factors, which would open the possibility of mechanistic studies to link signaling and chromatin.

We used a custom siRNA library targeting 160 chromatin factors (such as methyl-CpG-binding proteins, DNA modifying enzymes, lysine acetyltransferases and deacetylases, methyltransferases and demethylases; full list in Supplementary Table S4). As above, IMR90 cells were grown in wells, each well transfected with an siRNA pool targeting one specific factor, total RNA was extracted and tested by Nanostring analysis (Figure 1D). To evaluate the efficiency of the RNAi approach, we included in our Nanostring measurements 8 genes that were targeted by one of the siRNA pools (DNMT1, PPM1D, TET2, TET3, UHRF1, ZBTB4, ZBTB33, ZBTB38, Supplementary Figure S1C). Upon transfection of the specific siRNA, we observed a moderate depletion (∼2-fold) for two of the controls (TET2 and TET3), and a greater than 5-fold mRNA depletion for the remaining six controls (DNMT1, PPM1D, UHRF1, ZBTB4, ZBTB33, ZBTB38).

Having validated the approach, we next analyzed the expression of the 42 tissue-restricted genes in the ∼160 samples (control and transfected cells). The normalized data, presented in Figure 1E, show that only 21 of the ∼6700 probe/siRNA pairs tested show a *Z*-score >2.5.

For 25 genes out of 42, no RNAi gave an induction of *Z* >2.5. Thirteen genes out of 42 had a single hit with *Z* >2.5. Finally, just four genes out 42 had two hits with *Z* >2.5: ASZ1, DDX53, FMR1NB and ADAM12. ADAM12 was induced by depletion of the deacetylase SIRT6 (*Z*-score = 3.12, *P*-value = 9 × 10^−4^), and also by depletion of the acetyltransferase KAT2A (*Z*-score = 2.53, *P*-value = 5.7 × 10^−3^). Neither SIRT6 nor KAT2A were able to activate any of the other tissue-restricted genes.

The results of this screen therefore show that most tissue-restricted genes are refractory to induction by depletion of a single chromatin factor. Again, one exception to this rule is the gene ADAM12, for which expression is induced in both screens. We focused the rest of the work on this gene, in order to validate our findings, study their physiological relevance in the context of cancer, and clarify the interplay of signaling factors and chromatin regulators in its transcriptional regulation.

### The kinase activity of TAK1 is necessary for the induction of ADAM12

**Figure 2.**
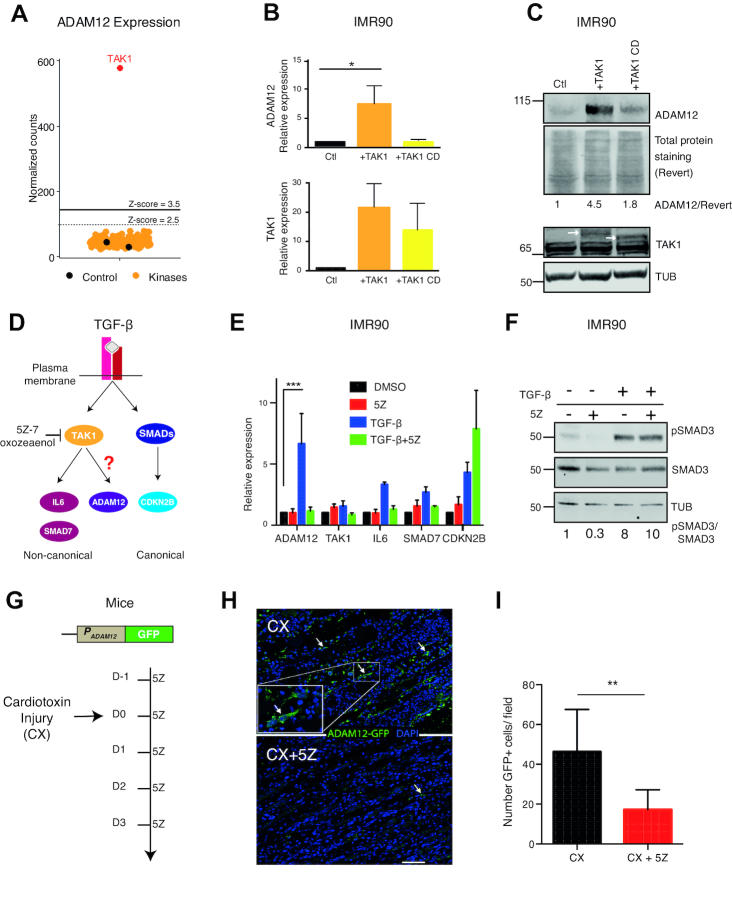
TAK1 mediates ADAM12 induction in vitro and *in vivo*. (**A**) Results of the signaling screen: only TAK1 expression resulted in the activation of ADAM12. Yellow and red dots represent ADAM12 normalized Nanostring counts in IMR90 infected with the kinase, each dot representing one kinase. Black dots represent ADAM12 expression level in control cells (infected with empty vector). (**B**) RT-qPCR validation of ADAM12 reactivation by TAK1 in IMR90 cells. ADAM12 was activated by the wild-type form of TAK1 but not by the catalytic dead form of TAK1 (TAK1 CD). (**C**) Western blot analysis of ADAM12 protein levels following the overexpression of wild-type and catalytic dead TAK1 (TAK1CD). Ctl: cells infected with the empty vector. The white arrows represent the exogenous TAK1; TAK1CD is lower because it does not self-phosphorylate. Tubulin (TUB) is the loading control. For ADAM12, concanavalin A enrichment was performed. (**D**) Schematic representation of canonical and non-canonical TGF-β pathways. TAK1 is a component of the non-canonical TGF-β pathway. (**E**) RT-qPCR on the indicated genes in the presence or absence of the TAK1 kinase inhibitor (5Z)-7-Oxozeaenol (5Z). IMR90 were pre-treated with 0.3μM 5Z or DMSO for two hours, followed by stimulation with 5 ng/ml of TGF-β for 6 h. ADAM12 induction by TGF-β is abolished by 5Z. (**F**) Control western blot showing the phosphorylation of SMAD3, indicating the activation of canonical TGF-β pathway even though TAK1 was inhibited by 5Z. (**G**) Testing the dependence of ADAM12 on TAK1 *in vivo*: reporter mice with the ADAM12 promoter (P_ADAM12_) driving GFP expression were subjected to an injury (cardiotoxin injection into the tibialis muscle). Some of the mice were treated by intraperitoneal injection of TAK1 inhibitor (5Z) at 5 mg/kg before and after injury, while the controls were injected only with solvent (DMSO). Three days after the injury, mice were sacrificed and muscle tissue was dissected. (**H**) Immunofluorescence on injured muscle shows that 5Z treatment reduces GFP induction. The arrows indicate GFP-positive cells. Scale bar: 150 μm. (**I**) Quantification of panel H (10 fields counted in each condition). All the experiments were performed at least three time except for ADAM12 western blot and mice experiments. The statistical analysis was performed with one way ANOVA followed by Dunnett's test, except for panel I where a Mann–Whitney test was performed. In all figures, we used the following conventions: *P < 0.05, **P < 0.01, ***P < 0.001, ****P < 0.0001.

As shown in Figure 2A, expression of ADAM12 was induced only by one kinase: TAK1. The ADAM12 mRNA induction was verified by RT-qPCR after an independent infection with the TAK1 expressing vector (Figure 2B); in contrast a point mutant of TAK1 devoid of kinase activity (TAK1 catalytic dead, or TAK1 CD), failed to induce ADAM12 expression. Wild-type TAK1, but not the CD mutant, also induced the ADAM12 protein, as shown by western blotting (Figure 2C). Similar results were also obtained in an other non-transformed human lung cell line, MRC5 (Supplementary Figure S2A).

### TAK1 links TGF-β to ADAM12 induction

TAK1 is activated by TGF-β (20), and more specifically it acts in the non-canonical branch of the TGF-β pathway, stimulating the transcription of genes such as IL-6 (Figure 2D). This non-canonical branch is distinct from the canonical branch, which involves SMADs, and leads to activation of genes such as CDKN2B (Figure 2D). It has also been shown that ADAM12 can be induced by TGF-β (21–25). However, to the best of our knowledge, the kinase mediating this activation has not been reported. Our previous results suggested that TAK1 could be a candidate.

To test this hypothesis, we first used a well-characterized chemical inhibitor of TAK1, (5Z)-7-oxozeaenol (40), referred to as ‘5Z’ hereafter. We treated IMR90 cells with TGF-β, in combination with 5Z or just its solvent, DMSO. As expected, TGF-β treatment (5 ng/ml, 6 h), induced all the target genes we examined (ADAM12, IL-6, SMAD7, CDKN2B; Figure 2E). Combining 5Z with TGF-β blocked the induction of ADAM12 and IL-6, whereas it did not affect CDKN2B induction, which was in fact potentiated. We also verified that 5Z treatment did not interfere with SMAD3 phosphorylation, a readout of the canonical pathway activation (Figure 2F); furthermore similar results were obtained in MRC5 cells (Supplementary Figures S2B and C).

The data obtained in cell culture strongly suggested that TAK1 mediated the induction of ADAM12 by TGF-β. To assess whether this was also the case in an *in vivo* setting, we used transgenic reporter mice in which GFP is driven by the ADAM12 promoter (Figure 2G). In these animals, wounding the muscle with cardiotoxin leads to the formation of ADAM12-positive myofibroblasts, which are marked by GFP, and this process depends on TGF-β (32). Treating mice with 5Z before and after injury led to a significant decrease of GFP+ cells in the regenerating muscle (Figures 2H and I). Consistent with a decrease in the number of GFP+ cells, the ADAM12 protein was also decreased in muscle lysates, as measured by western blot, when TAK1 was inhibited (Supplementary Figures S2D and E).

Altogether these data show that TAK1 activation can induce ADAM12 expression in normal cells. In addition, TAK1 activity is necessary to induce ADAM12 expression *in vitro* and *in vivo*, in a mouse injury model.

### An RNAi screen identifies chromatin modifiers that regulate ADAM12 expression

**Figure 3.**
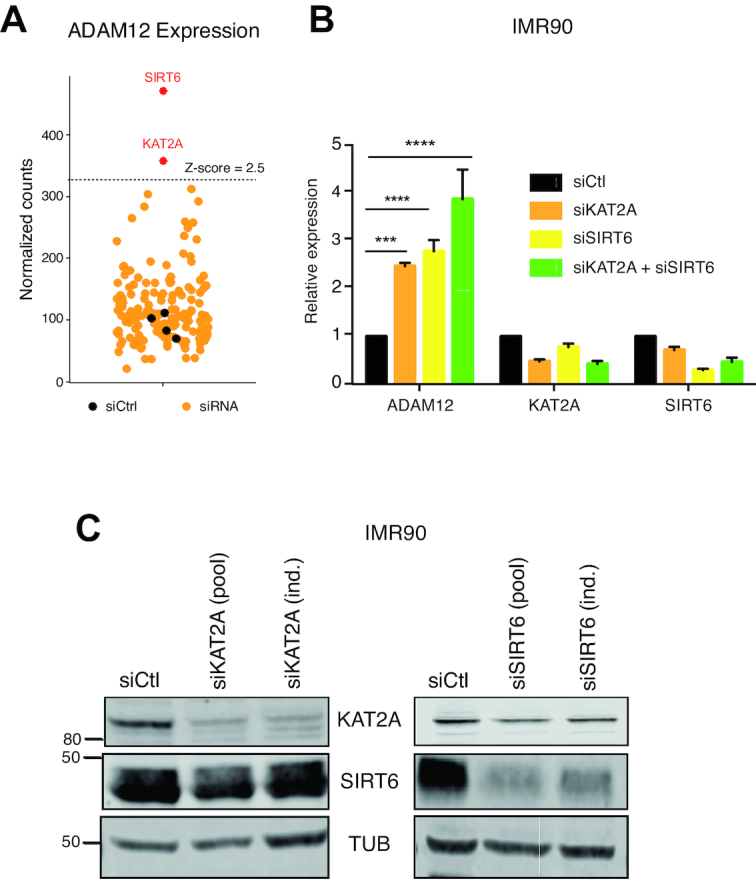
KAT2A and SIRT6 repress ADAM12 expression in normal cells. (**A**) Results of the chromatin screen: knockdown of KAT2A or SIRT6 causes the reactivation of ADAM12. Yellow dots represent ADAM12 normalized Nanostring counts in IMR90 transfected with targeting siRNAs; black dots for cells transfected with a non-targeting siRNA (siCtrl). (**B**) RT-qPCR representing relative expression of ADAM12, KAT2A and SIRT6 in IMR90 cells infected with siCtl (non-targeting siRNA), siKAT2A and/or siSIRT6. These siRNAs are independent from those used in the screen. Statistical analysis was performed with a one-way ANOVA followed by a Dunnett's test. (**C**) Western blot analysis showing efficient down regulation of KAT2A and SIRT6 after transfection of either the original pool of siRNA used in the screen (pool), or of individual siRNAs (ind.).

The top two siRNA targets causing reactivation of ADAM12 were SIRT6 and KAT2A (formerly known as GCN5, Figure 3A). The results were validated with an additional siRNA not present in the initial pool (Figures 3B and C), and similar observations were made in MRC5 cells (Supplementary Figures S3A–C). Notably, combined knockdown of the two genes led to higher ADAM12 induction than either individual knockdown, suggesting they might act in different pathways (Figure 3B).

SIRT6 is a well-known transcriptional repressor that acts in part by deacetylating histones at H3K9 (41). In contrast, KAT2A generally mediates transcriptional activation, by acetylating lysines, including H3K9, as part of the SAGA complex (42). Therefore, it was unexpected that knocking down KAT2A, an activator, led to ADAM12 induction, and thus we chose to investigate further this surprising finding.

### KAT2A acts upstream of TAK1 and interacts with TAK1

KAT2A is known to act on certain non-histone proteins, such as the kinase PLK4 (43). Therefore we considered the hypothesis that KAT2A might act upstream of TAK1, and negatively control its activity, which could account for the induction of ADAM12 upon KAT2A knockdown.

**Figure 4.**
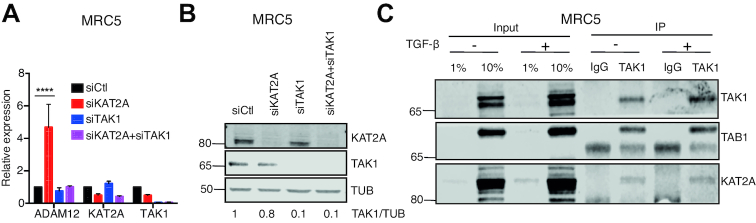
Epistasis and interaction between KAT2A and TAK1. (**A**) RT-qPCR on the indicated genes in MRC5 cells transfected with siCtl (non-targeting siRNA), siKAT2A and/or siTAK1. ADAM12 is induced by the knockdown of KAT2A but this is abolished by the simultaneous knockdown of TAK1. Statistical analysis was performed with a two-way ANOVA followed by a Dunnett's test. (**B**) Western blot showing expression level of TAK1 and KAT2A after siRNA transfection. (**C**) Co-immunoprecipitation of endogenous TAK1 from MRC5 cells with or without stimulation by 5 ng/ml TGF-β revealed interaction with KAT2A. TAB1, a known interactor of TAK1, served as a positive control.

To test this idea, we first performed TAK1 knockdown simultaneously with KAT2A knockdown. In that situation, depletion of KAT2A failed to induce ADAM12 (Figures 4A and B). In an independent approach, we also pre-treated cells with 5Z and then performed KAT2A knockdown; this also dampened ADAM12 induction (Supplementary Figures S4A and B).

The result of these two experiments shows that removal of KAT2A does not induce ADAM12 when TAK1 is absent or inactive. This result is compatible with the hypothesis that KAT2A functions upstream of TAK1 and negatively regulates its activity. As KAT2A can physically interact with certain non-histone proteins, we asked whether it might interact with TAK1. Immunoprecipitation of endogenous TAK1 was performed on MRC5 cells using the known cofactor TAB1 as a positive control (44). We also detected KAT2A in TAK1 immuno-precipitates (Figure 4C). The interaction pre-existed before TGF-β addition, and persisted after TGF-β addition (Figure 4C).

### TAK1 drives ADAM12 expression in a triple-negative breast cancer cell line

After establishing that TAK1 mediates ADAM12 induction by TGF-β in normal human cells, we asked whether it was also involved in the sustained expression of ADAM12 seen in tumor cells, and particularly in Triple-Negative Breast Cancer (TNBC) cells.

**Figure 5.**
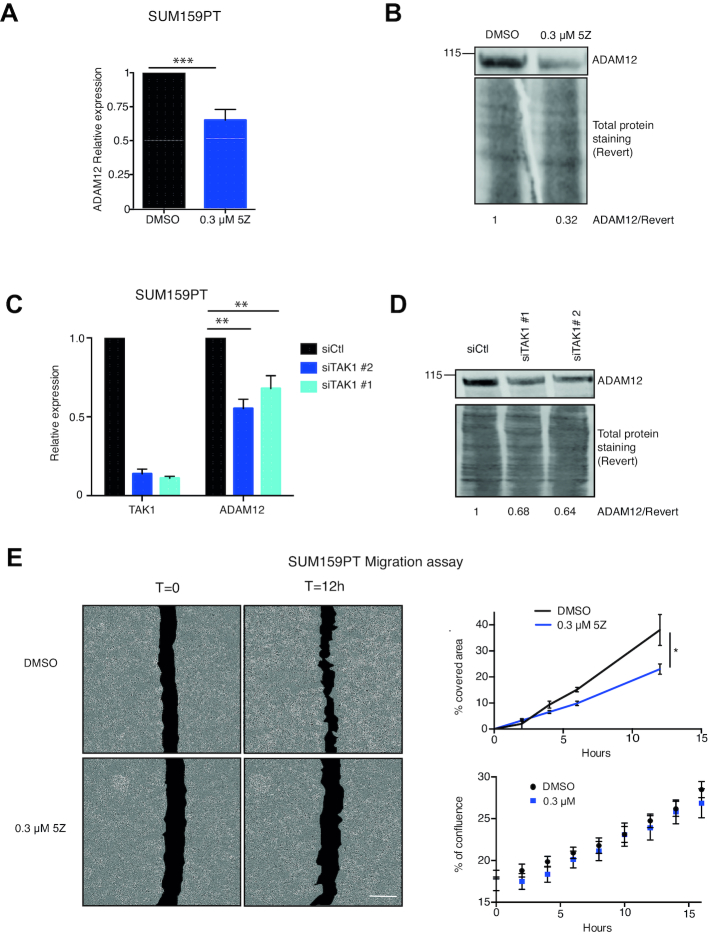
TAK1 is involved in ADAM12 expression in breast cancer cells. (**A**) SUM159PT breast cancer cells were treated with 0.3 μM or 1 μM 5Z for four days and the level of ADAM12 was assessed by RT-qPCR. Inhibition of TAK1 by 5Z reduced the level of ADAM12 transcripts. (**B**) Western blot analysis showing ADAM12 protein expression after 5Z treatment of SUM159PT cells. Concanavalin A enrichment was performed. **(C)** RT-qPCR showing the expression levels of TAK1 and ADAM12 in SUM159PT cells transfected with non-targeting control siRNA (siCtl) or siTAK1 for four days. Knockdown of TAK1 decreased the levels of ADAM12 transcripts. (**D**) Western blot showing ADAM12 expression after knockdown by siCtl or siTAK1 in SUM159PT cells. As in panel B. (**E**) Wound healing assay. SUM159PT cells were treated with 0.3 μM and 1 μM 5Z for 4 days, following treatment, a scratch was made in the dishes and it was imaged every 2 h using the Incucyte live cell system. The area of the wound at different time points was measured by ImageJ software, then a percentage of the area covered by time was determined and is plotted in the panel on the right. Inhibition by 5Z delays the wound healing process. The statistical analysis was performed employing a two way ANOVA test followed by a Dunnett's test. Bottom right panel: growth rate of the cells measured as their percentage of confluence after seeding in a non-scratched plate; 5Z does not affect the growth rate.

For this we used SUM159PT cells, which express ADAM12 (19). We first inhibited TAK1 chemically, using 5Z; this led to a decrease of ADAM12 mRNA and protein expression (Figures 5A and B), an effect also seen for another TNBC line, BT549 (Supplementary Figures S5A and B). We also knocked down TAK1 using two independent siRNAs in SUM159PT; this resulted in decreased ADAM12 expression as well (Figure 5C and D). Finally we found that, as in non-transformed cells, TAK1 interacts with KAT2A in SUM159PT cells (Supplementary Figure S5C).

The next question we sought to address was whether this molecular pathway could be shown to have phenotypical consequences. One of the known effects of ADAM12 is to promote cellular migration (16–18), so we tested this phenotype. We measured cell migration quantitatively, and found that treating SUM159PT cells with 5Z decreased their migration in a ‘scratch assay’ (Figure 5E). In parallel experiments, we quantified the effect of 5Z on the growth rate of cells after seeding in a non-scratched plate (Figure 5E). The growth of solvent-treated and 5Z-treated cells was identical, ruling out decreased proliferation as the cause of decreased migration.

### KAT2A represses ADAM12 expression in a triple-negative breast cancer cell line

**Figure 6.**
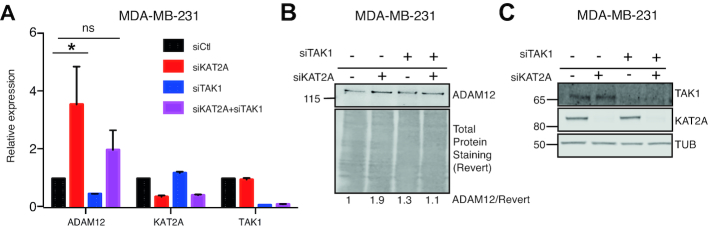
KAT2A negatively regulates ADAM12 expression in breast cancer cells. (**A**) RT-qPCR showing the relative expression level of ADAM12, TAK1 and KAT2A in MDA-MB-231 cells transfected with non-targeting control siRNA (siCtl), siKAT2A, siTAK1 and/or siKAT2A for four days as depicted. The statistical analysis was performed with a two-way ANOVA test followed by a Dunnett's test. (**B**) Western blot showing ADAM12 expression after siRNA transfection in MDA-MB-231. For ADAM12, concanavalin A enrichment was performed. (**C**) Validation of siRNA efficiency by western blotting.

Next, we asked if our observation that KAT2A restricts ADAM12 expression, made in a non-transformed cell context, was also relevant in cancer. For this we used a different TNBC line, MDA-MB-231, in which the expression of ADAM12 is modest compared to SUM159PT (45). In this context as well, knockdown of KAT2A led to an increased level of ADAM12 mRNA (Figure 6A) and protein (Figure 6B). Importantly, this effect was not seen when TAK1 was simultaneously knocked down (Figures 6A–C). This establishes that the KAT2A/TAK1 regulatory axis is also functional in TNBC lines. Finally, we verified that the TAK1/KAT2A interaction was also detectable in MDA-MB-231 cells, both in the cytoplasmic and nuclear compartments (Supplementary Figure S6).

### The analysis of human cancer expression data supports the regulation of ADAM12 by TAK1 and KAT2A in various tumor types

Lastly, we sought to determine whether our findings on normal or transformed cells represent a general mechanism. For this, we performed a bioinformatic analysis of TCGA data, starting with breast cancer.

**Figure 7.**
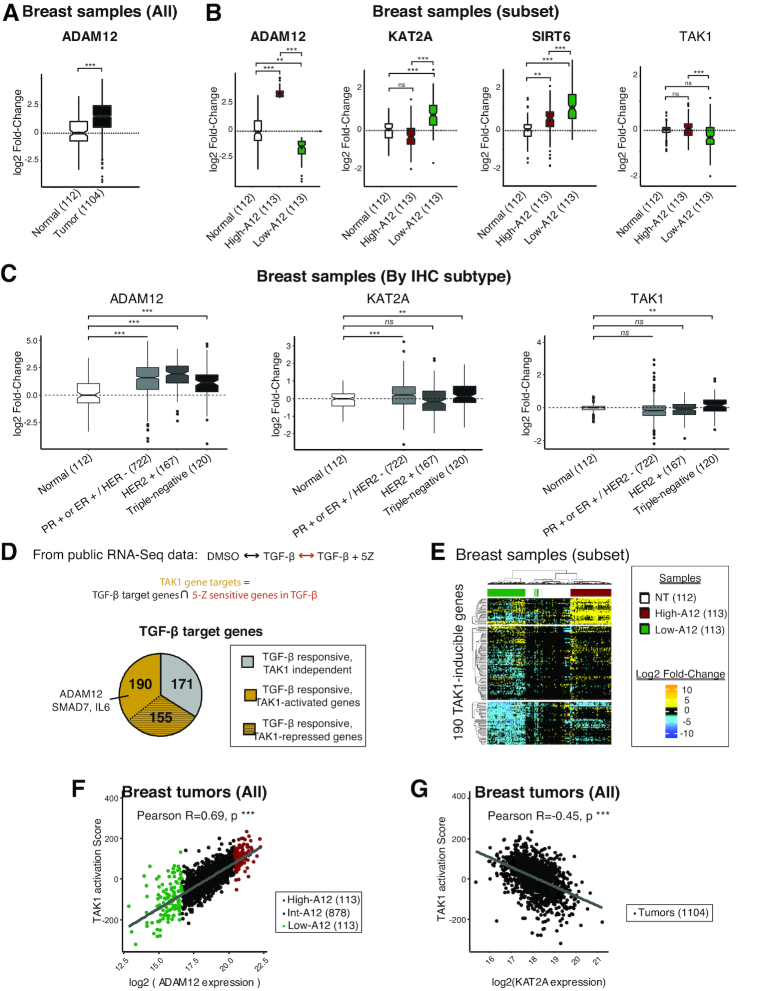
TCGA analysis revealed co-expression of ADAM12 with KAT2A, TAK1 and TAK1-inducible genes signature in breast tumors. (**A**) ADAM12 expression in breast tumors and normal breast tissue from the TCGA database. The boxplots contain 50% of the values, with a notch at the median value, and a diamond at the average value. The whiskers depict the first and last quartiles, and outliers appear as black dots. (**B**) ADAM12, KAT2A, and TAK1 expression in normal breast tissues and a subset of breast cancer samples from the TCGA database, selected based on their low (first decile, green) or high (ninth decile, red) ADAM12 expression. (**C**) ADAM12, KAT2A, and TAK1 expression in normal breast tissues and breast cancer samples from the TCGA database stratified by subtype. (**D**) Definition of the TAK1 signature, using transcriptomic data for primary cells treated with TGF-β with or without the TAK1 inhibitor 5Z. The TAK1 signature contains the genes that are upregulated by TGF-β addition, but not upregulated in the TGF-β+5Z condition. (**E**) Hierarchical clustering with euclidean distance metric and Ward's linkage method of highest and lowest ADAM12 expressing tumors samples, and normal breast tissue samples. The unsupervised clustering of breast tumors according to the TAK1 signature almost perfectly segregates tumors according to ADAM12 expression. (**F**) TAK1 activation score (calculated from the expression of genes in the TAK1 signature) correlates positively with ADAM12 expression in 1104 breast tumors. The high-ADAM12 samples are shown in red and low-ADAM12 in green. The statistical analysis was performed with a two-way ANOVA test, followed by a Tukey HSD test (*** denotes *P* < 0.001). (**G**) TAK1-activation score negatively correlates with KAT2A expression in 1104 breast tumors. Statistics as in panel E.

We first verified the previously described observation that ADAM12 is upregulated in breast tumors (Figure 7A). We then stratified the breast tumor samples based on ADAM12 mRNA levels, and compared the high-ADAM12 group (highest decile for ADAM12 expression) to the low-ADAM12 group (lowest decile) and to normal tissue (Figure 7B). We found that low ADAM12 expression was associated with high KAT2A expression (Figure 7B). This association is compatible with the model we have put forward based on our mechanistic work with cell lines. Conversely, TAK1 was more expressed in high-ADAM12 tumors than in low-ADAM12 tumors (Figure 7B), again consistent with our in vitro work. When the breast tumors were stratified by subtype, the analysis revealed that not only triple-negative tumors contained high-ADAM12 and low-TAK1 samples, but the other groups did as well (Figure 7C).

The mRNA level of TAK1 could be an imperfect proxy of its activity, thus to strengthen these data, we performed a more detailed analysis, which started by identifying a ‘TAK1 signature’ (Figure 7D). For this we used transcriptome data obtained in primary human cells, treated with TGF-β, 5Z or TGF-β+5Z (42). We identified 516 TGF-β responsive genes, which we divided into three classes. The first class contains 171 genes that are induced by TGF-β equally well in the presence or absence of 5Z and are therefore TAK1-independent. The second class contains 155 genes, including SMAD3, that are induced by TGF-β more strongly when 5Z is present; those may be repressed directly or indirectly by TAK1. The last class of genes are induced by TGF-β less strongly when 5Z is present, and are therefore potentially dependent on TAK1. For further analysis, we focused on this group of 190 genes (including ADAM12, SMAD7, and IL-6), referred to as a ‘TAK1 signature’ (Figure 7D and Supplementary Table S7).

To test its discriminative power relative to ADAM12 expression, we assembled a set of 338 TCGA breast samples (112 normal, 113 high-ADAM12, and 113 low-ADAM12), and performed unsupervised clustering based on the TAK1 signature. This resulted in near-perfect segregation of normal, ADAM12-low, and ADAM12-high breast tumors (Figure 7E). Therefore TAK1 activity is indeed strongly associated with ADAM12 expression in breast tumors.

This first analysis had been performed on a highly contrasted group of tumors (highest versus lowest ADAM12 expression), so we lastly asked whether it held true for the rest of the tumors as well. For this, we used as a metric the ‘TAK1 score’, which reflects the expression of the 190 genes in the TAK1 signature (see materials and methods).

Using all 1104 tumors in the dataset, we observed a clear correlation between the TAK1 activation score and the ADAM12 expression level (Pearson's *r* = 0.69; Figure 7F). Conversely, the TAK1 activation score is significantly anti-correlated with the KAT2A expression level (Pearson's *r* = –0.45; Figure 7G). A thousand random samplings failed to yield a set of 190 genes that displayed these correlations (Supplementary Figure S7A), supporting the validity of the TAK1 signature.

Finally, we sought to determine whether our findings may apply to cancer types other than breast malignancies. We repeated the same bioinformatic analyses on 1044 lung tumors (Supplementary Figures S7B–D), and on 505 colon adenocarcinomas (Supplementary Figures S7E–G). In both tumor types we found correlations between high ADAM12 expression and lower KAT2A levels (Supplementary Figures S7C and F). A high TAK1 signature very clearly separated high-ADAM12 from low-ADAM12 tumors both in lung (Supplementary Figure S7D) and colon (Supplementary Figure S7G), and the TAK1 activation score was positively correlated with ADAM12 expression, and negatively correlated with KAT2A expression, in both tumor types (Supplementary Figures S7H–I).

Altogether, these data agree with and extend our mechanistic data obtained in vitro, arguing that KAT2A negatively regulate ADAM12 expression in several prevalent human tumor types. We also conclude that TAK1 activity is a strong positive determinant of ADAM12 expression in lung, breast, and colon cancer.

## DISCUSSION

### Single hits affecting signaling pathways or chromatin regulation are generally not sufficient to activate tissue-restricted genes

We tested ∼200 activated kinases for their capacity to induce tissue-restricted gene expression in non-transformed cells. While TAK1 could robustly activate ADAM12 in normal lung cells, we found no other instance of a tissue-restricted gene being strongly reactivated by a single activated kinase. The system we used has several possible limitations that need to be considered when interpreting this result. First, the kinases were genetically activated by the inclusion of a myristoylation signal, which causes recruitment of the kinases to the cell membrane. This could potentially lead to false negative results if a given kinase needs to be in a different cellular compartment, for instance the nucleus, to activate gene expression. Second, we have no formal proof that each single kinase was functional, but this collection has been validated in several previous publications (26,39,46–49), and we verified in our system that each kinase was expressed. We also verified the ability of the probes to detect an induction, even small.

Similarly, we tested 160 siRNA pools directed against chromatin modulators for their capacity to induce expression of the tissue-restricted genes. Again, most genes were refractory to this treatment, with one of the rare exceptions being ADAM12.

The tissue-restricted genes are embedded in repressive chromatin in non-expressing cells (10). For the illegitimate expression to take place, this chromatin has to become permissive, and it is possible that cell division helps this process, as chromatin is remodeled along with DNA replication (50). In the course of our genetic screens (4 or 5 days, see Figure 1), the IMR90 cells divide ∼3 times, and we have found that these conditions are appropriate to identify a negative regulator of a non-tissue restricted gene (27). However, we cannot rule out the possibility that longer incubations with the activated kinases or the siRNAs would lead to increased chromatin remodeling and additional tissue-restricted genes being reactivated.

We can conclude, however, that ADAM12 is more readily reactivatable than the other genes tested. This property does not seem to apply to all placenta-specific genes as XAGE3, which was also present in our gene set, was never induced in our screens. Therefore, ADAM12 likely undergoes a specific regulation.

### ADAM12 is repressed by SIRT6: possible mechanisms

One of the negative regulators of ADAM12 expression identified in our siRNA screen is the histone deacetylase SIRT6. Interestingly, SIRT6 is often underexpressed in tumors, and its loss contributes to cellular transformation (51); a corresponding induction of ADAM12 could possibly contribute to this phenotype. Mechanistically, SIRT6, as several other sirtuins, has the capacity to repress gene promoters by causing histone deacetylation (41). Therefore a simple hypothesis is that SIRT6 acts directly on the ADAM12 promoter and keeps its expression low. ChIP-Seq has been performed on SIRT6 in mouse embryonic fibroblasts (52), and these data suggest that the ADAM12 promoter is directly bound by SIRT6, at least in this cell type. Other non-exclusive possibilities might also occur: SIRT6 could repress the expression of an ADAM12 activator. Finally, we note that SIRT6 has been shown to bind and activate KAT2A in certain contexts (53), therefore this might also contribute to ensuring the repression of ADAM12 by SIRT6.

### TAK1 links TGF-β to ADAM12 induction: pathways and consequences

The kinase MAP3K7/TAK1 was a strong outlier in our signaling screen, as it potently activates the expression of ADAM12 even in lung cells. There have been several indications that TGF-β can induce ADAM12 expression (21–25). Now we bring evidence that the non-canonical branch of TGF-β signaling is especially involved, via the involvement of TAK1. Several molecular pathways are activated downstream of TAK1 (54), one of which is NF-kB (55–57). As NF-kB has been shown to upregulate ADAM12 in response to TGF-β (24), it may constitute the next element in the signalling cascade linking TGF-β, TAK1 and ADAM12 induction.

ADAM12 has been shown to contribute positively to tumorigenesis (17,19,58), therefore its extinction is predicted to have beneficial effects. TAK1 inhibitors have been actively investigated in the context of immunity, fibrosis, and cancer (59–62). Our results suggest that these molecules could be particularly relevant in ADAM12-positive tumors. Based on our TCGA analysis, these represent a large number of potentially targetable cases.

### A repressive role for KAT2A

We report that depletion of KAT2A increases TAK1 expression in multiple cellular contexts. This result is counterintuitive given that KAT2A, a core component of the SAGA complex, is a global activator of PolII transcription (63). Our experiments with chemical inhibitors and RNAi show that KAT2A depletion has no effect on ADAM12 expression when TAK1 is absent or inactive. The simplest interpretation of these findings is that KAT2A inhibits the activity of TAK1, and an indication that this might occur is our finding that KAT2A and TAK1 co-immunoprecipitate. Future work will determine whether KAT2A affects TAK1 protein abundance, localization, or activity. Such a mechanism has recently been demonstrated for the Polo-Like Kinase PLK4, the activity of which is inhibited by KAT2A-mediated lysine acetylation (43).

### Illegitimate gene expression in cancer

Our work using normal cells show that most tissue-restricted genes are more resistant to activation signals than ADAM12. This may reflect the existence of several layers of epigenetic repression, for instance repressive histones coupled to DNA methylation, which would require more than one event to be relieved. Two predictions from this hypothesis are that ADAM12 should be reactivated in more tumors than the other tissue-restricted genes, and also that the expression of tissue-restricted genes may be a rather late event during oncogenesis, occurring only after several transforming events have accumulated. If this prediction is correct, then immunotherapy on illegitimately expressed genes would be of limited efficacy on tumors in their early stages.

## DATA AVAILABILITY

The Nanostring data have been deposited in GEO under reference GSE124101.

## Supplementary Material

Supplementary Data
