# Supplementary material for: Genetic screens reveal mechanisms for the transcriptional regulation of tissue-specific genes in normal cells and tumors
Source: Nucleic Acids Res. 2019 Feb 7;47(7):3407–21. doi: 10.1093/nar/gkz080 (PMC6468300; doi:10.1093/nar/gkz080)
Supplement: Supplementary Data [file gkz080_supplemental_file.pdf]

Supplemental material for Naciri et al.

"Genetic screens reveal mechanisms for the transcriptional regulation of tissue-specific genes in normal cells and tumors"

Contents :

Supplemental Figure Legends
Supplemental Table Legends
Supplemental Figures S1-S7
Supplemental Tables 1-7

Supplemental Figure Legends

Supplemental Figure 1, Related to Figure 1.

(A) Validation of Nanostring probes with positive controls. The probes for each of the 42 tissue-restricted genes were tested on primary IMR90 cells (black dots), the transformed derivative SW39 cells (green dots), and on a positive control mRNA extracted from 10 mixed human cancer cell lines. The dotted line represents a 2-fold change over control. **(B)** Verification of kinase expression in the signaling screen: Nanostring counts of the Myr-Flag tag in non-infected cells (IMR90 or its transformed derivative SW39), and in cells infected by each of the 192 activated kinases (yellow dots). **(C)** Validation of siRNA efficiency on eight selected genes. Nanostring probes were used to measure the abundance of mRNA for each of the 8 indicated genes after transfection of siRNA pools targeting the gene in question (yellow bars), or transfection of non-targeting siRNA pools (green bars). Non-transfected cells are also included (black bars).

Supplemental Figure 2, Related to Figure 2.

(A) Relative expression of ADAM12 and TAK1 in MRC5 primary lung fibroblasts after infection with viral vectors expressing either wild-type TAK1 or the catalytic dead TAK1 (TAK1 CD). The statistical analysis was performed with a one way ANOVA followed by a Dunnett's test except for mice experiment where Mann Whitney was performed (* $p < 0.05$, ** $p < 0.01$, *** $p < 0.001$, **** $p < 0.0001$). **(B)** RT-qPCR representing the relative expression of TGF-β target genes in the presence or absence of TAK1 kinase inhibitor 5Z. MRC5 cells were pre-treated with 0.3μM 5Z or DMSO for two hours, followed by stimulation with 5ng/mL TGF-β for six hours. **(C)** Western blot showing the phosphorylation of SMAD3 in MRC5 even under 5Z treatment. Tubulin (TUB) served as a loading control. **(D)** Western blotting on ADAM12 in whole cell extracts of tibialis muscles after the indicated treatments. CD71 is a loading control that underwent Concanavalin A enrichment, like ADAM12. MW: lane with the molecular weights. **(E)** Quantification of panel D was performed using Image Studio lite (Licor) with CD71 as a loading control. All experiments have been performed three times while mice experiments were conducted twice.

Supplemental Figure 3, Related to Figure 3.

(A) RT-qPCR showing relative expression of ADAM12, KAT2A and SIRT6 in MRC5 cells transfected with siCtl (non-targeting siRNA), siKAT2A and/or siSIRT6 as indicated. These siRNAs are independent from the ones used in the screen. The statistical analysis was

performed employing a two-way two tailed ANOVA test followed by Dunnett's test. **(B)** Western blot analysis showing down regulation of KAT2A and SIRT6 after siRNA transfection in MRC5, Tubulin (TUB) was used as a loading control. **(C)** Western blot showing induction of the ADAM12 protein after siRNA against the indicated factors. All experiments were performed three times or more.

Supplemental Figure 4, Related to Figure 4.

Primary lung fibroblast IMR90 **(A)** and MRC5 **(B)** were transfected with siCtrl or siKAT2A and treated every day with 0.3µM 5Z or DMSO for 3 days. RT-qPCR shows relative expression of ADAM12 and KAT2A. The statistical analysis was performed with a one way ANOVA test followed by a Dunnett's test. All experiments were performed three times or more.

Supplemental Figure 5, Related to Figure 5.

(A) RT-qPCR showing relative expression of ADAM12 after treatment of BT549 cells with the TAK1 inhibitor 5Z at 0.3 µM concentration for 4 days. The statistical analysis was performed with a one way two ANOVA test followed by a Dunnett's test. **(B)** Western blot showing ADAM12 expression after 5Z treatment in BT549. For ADAM12, concanavalin A enrichment was performed. **(C)** Co-immunoprecipitation of endogenous TAK1 from SUM159PT after cellular fractionation revealed interaction with KAT2A in the cytoplasm and the nucleus. TAB1, co-factor of TAK1 served as a positive control. Histone H3 was used as a marker of the nuclear compartment, and tubulin as a marker of the cytoplasmic compartment.

Supplemental Figure 6, Related to Figure 6.

Co-immunoprecipitation of endogenous TAK1 from MDA-MB-231 cell line reveals interaction with KAT2A.

Supplemental Figure 7, Related to Figure 7.

(A) Random sampling procedure to test the validity of the TAK1 signature. **(B)** ADAM12 expression in lung tumors and normal lung from the TCGA database. The boxplots contain 50% of the values, with a notch at the median value, and a diamond at the average value. The whiskers depict the first and last quartiles, and outliers appear as black dots. **(C)** ADAM12, KAT2A, TAK1 expression in normal lung tissue and a subset of lung tumors, selected based

on their low (1st decile, green) or high (9th decile, red) ADAM12 expression. **(D)** Unsupervised clustering of lung tumors according to the TAK1 signature almost perfectly segregates tumors according to ADAM12 expression. **(E)** ADAM12 expression in colon cancer samples and normal colon tissue samples from the TCGA database. **(F)** ADAM12, KAT2A, TAK1 expression in normal colon tissue and a subset of colon tumors, selected based on their low (1st decile, green) or high (9th decile, red) ADAM12 expression. **(G)** Unsupervised clustering of colon tumors according to the TAK1 signature almost perfectly segregates tumors according to ADAM12 expression. **(H)** TAK1-activation score versus ADAM12 or KAT2A mRNA expression in lung cancer samples from TCGA database. The statistical analysis was performed employing a one way ANOVA test, following by a Tukey HSD test (*** denotes $p < 0.001$). **(I)** As in panel H, but on colon tumors.

Supplemental Table Legends

Table S1: List of 192 activated kinases used in the signaling screen.

Table S2: List and target sequence of 72 Nanostring probes.

Table S3: List and characteristics of 42 tissue-restricted genes analyzed in the screens.

Table S4: List of 160 chromatin regulators targeted in the siRNA screen.

Table S5: Sequence of qRT-PCR primers used.

Table S6: Sequence of siRNAs used.

Table S7: List of 190 genes constituting the TAK1 signature.

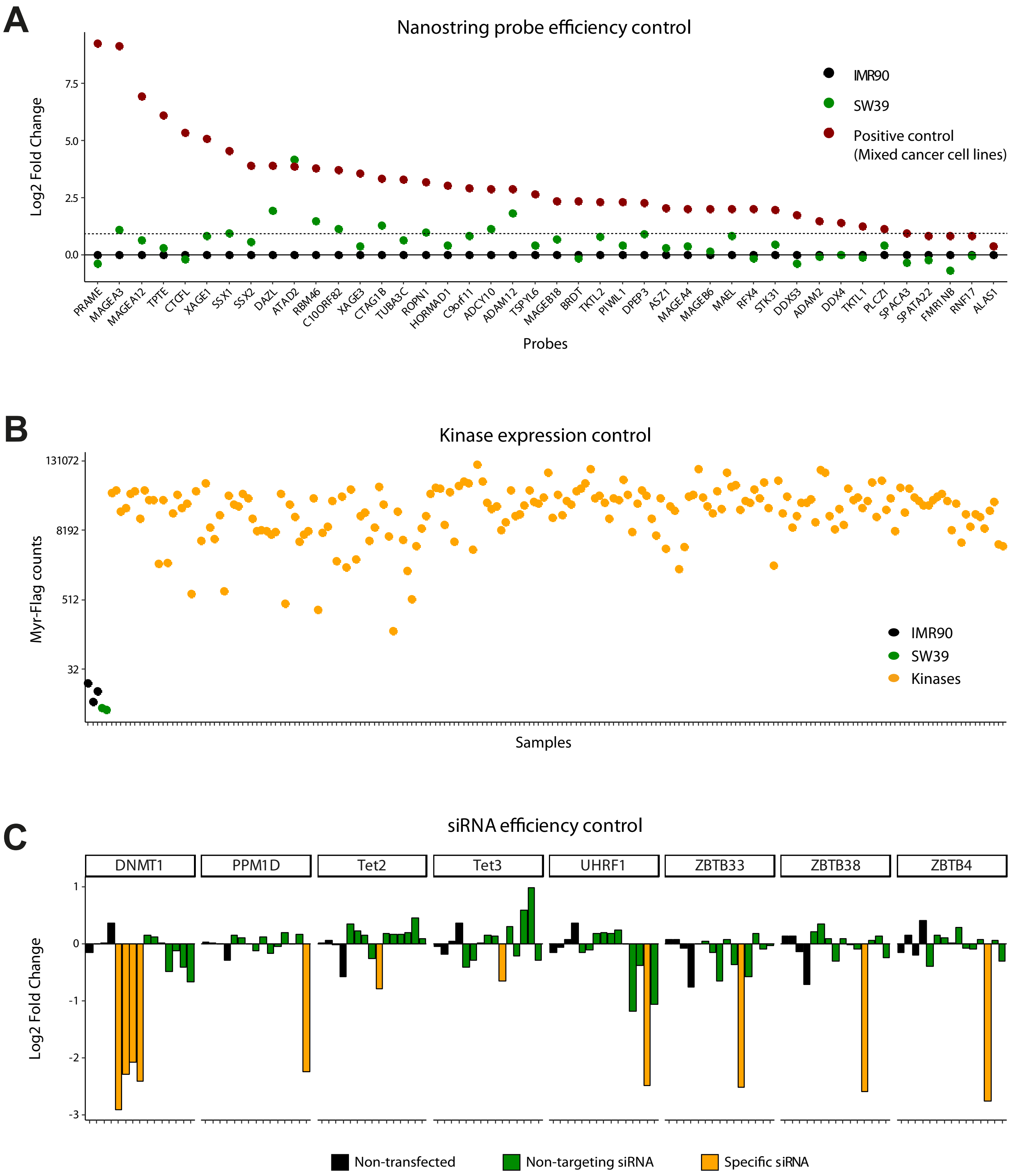

Figure S1
Naciri et al.

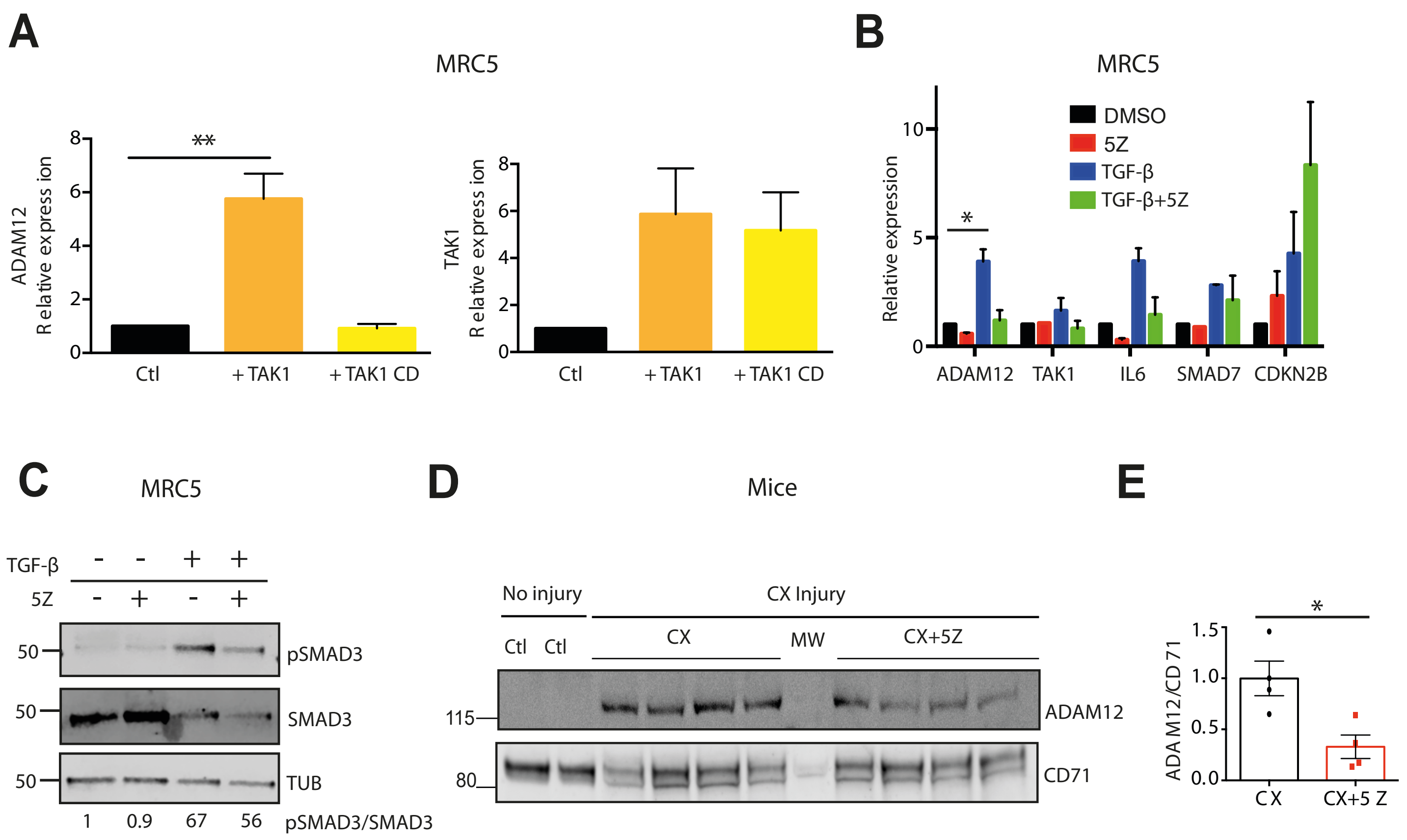

Figure S2
Naciri et al.

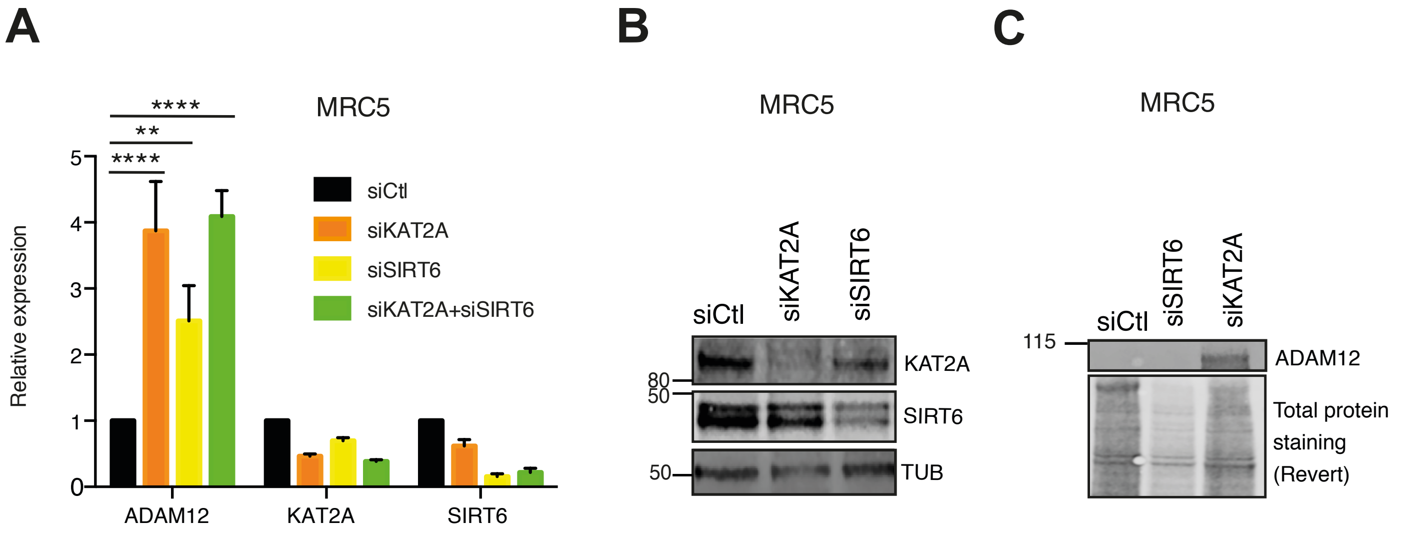

Figure S3
Naciri et al.

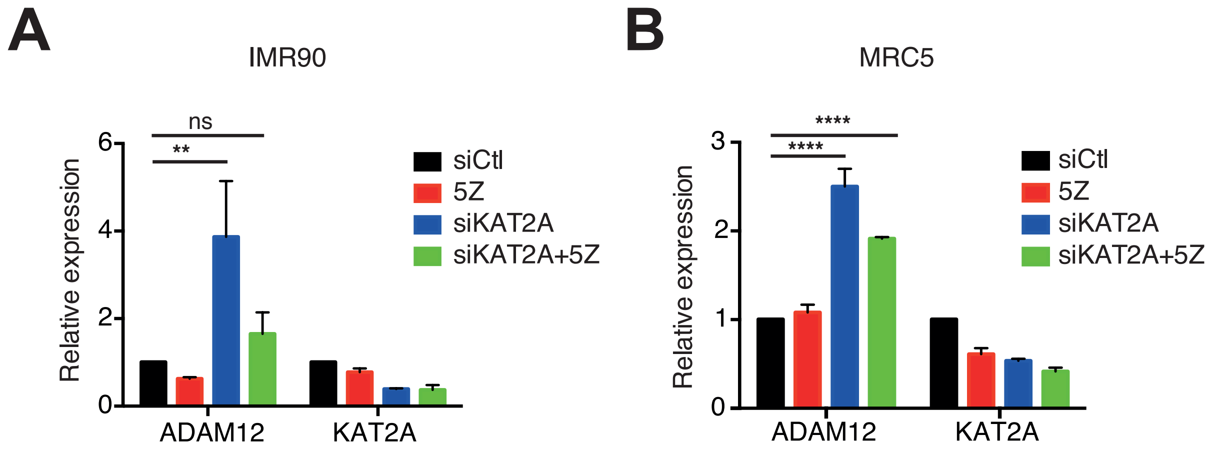

Figure S4
Naciri et al.

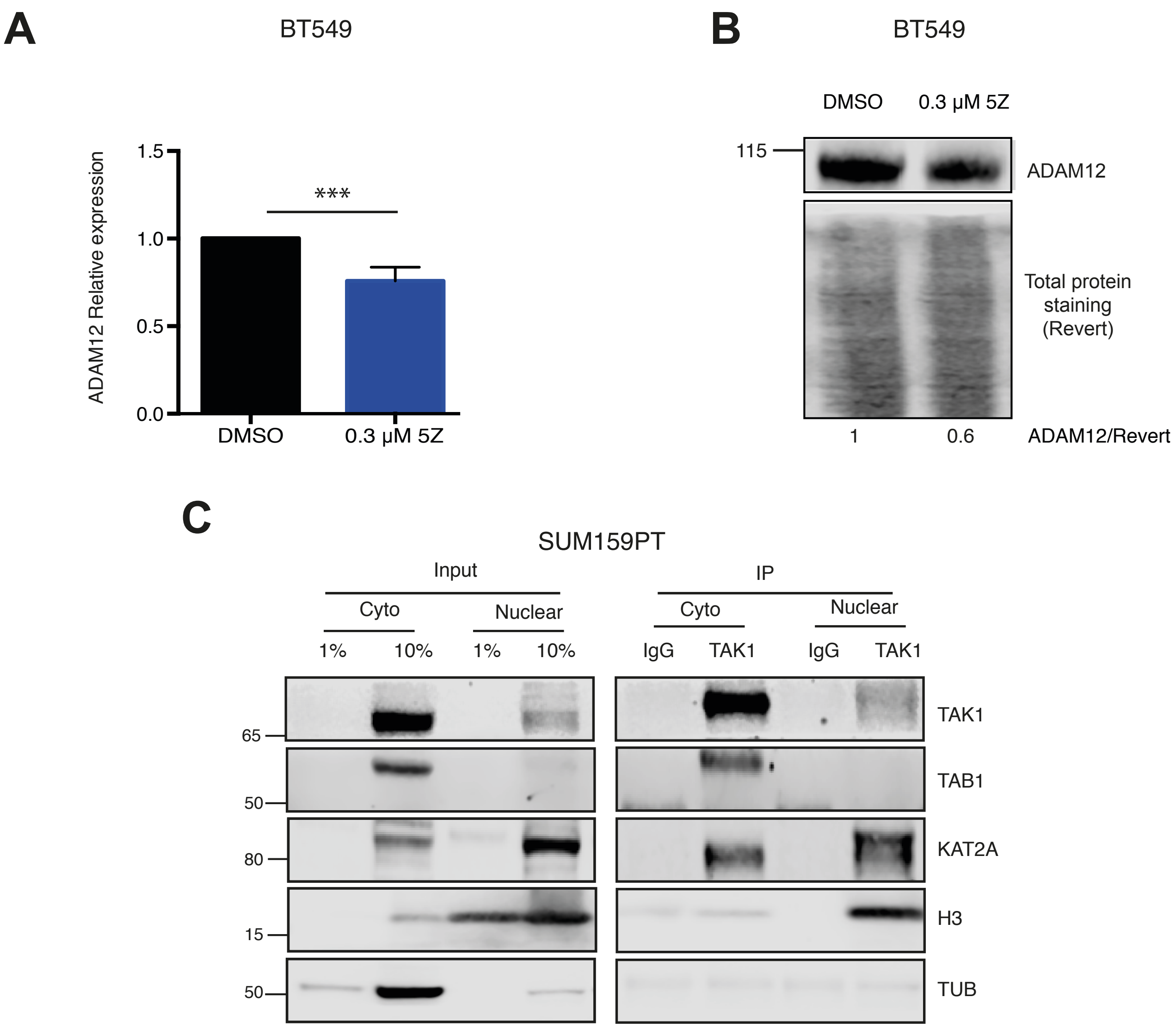

Figure S5
Naciri et al.

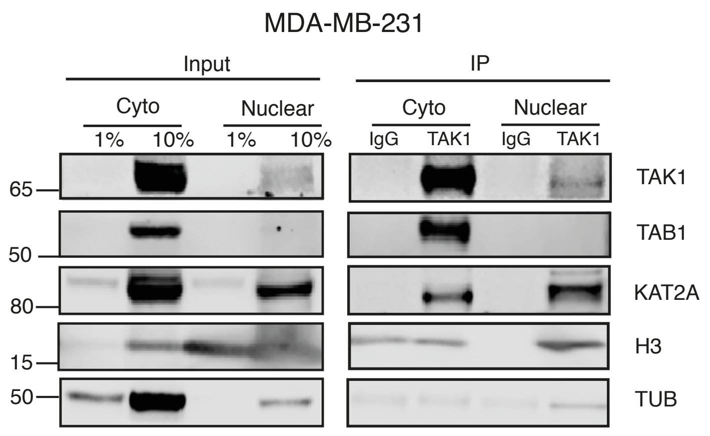

Figure S6
Naciri et al.

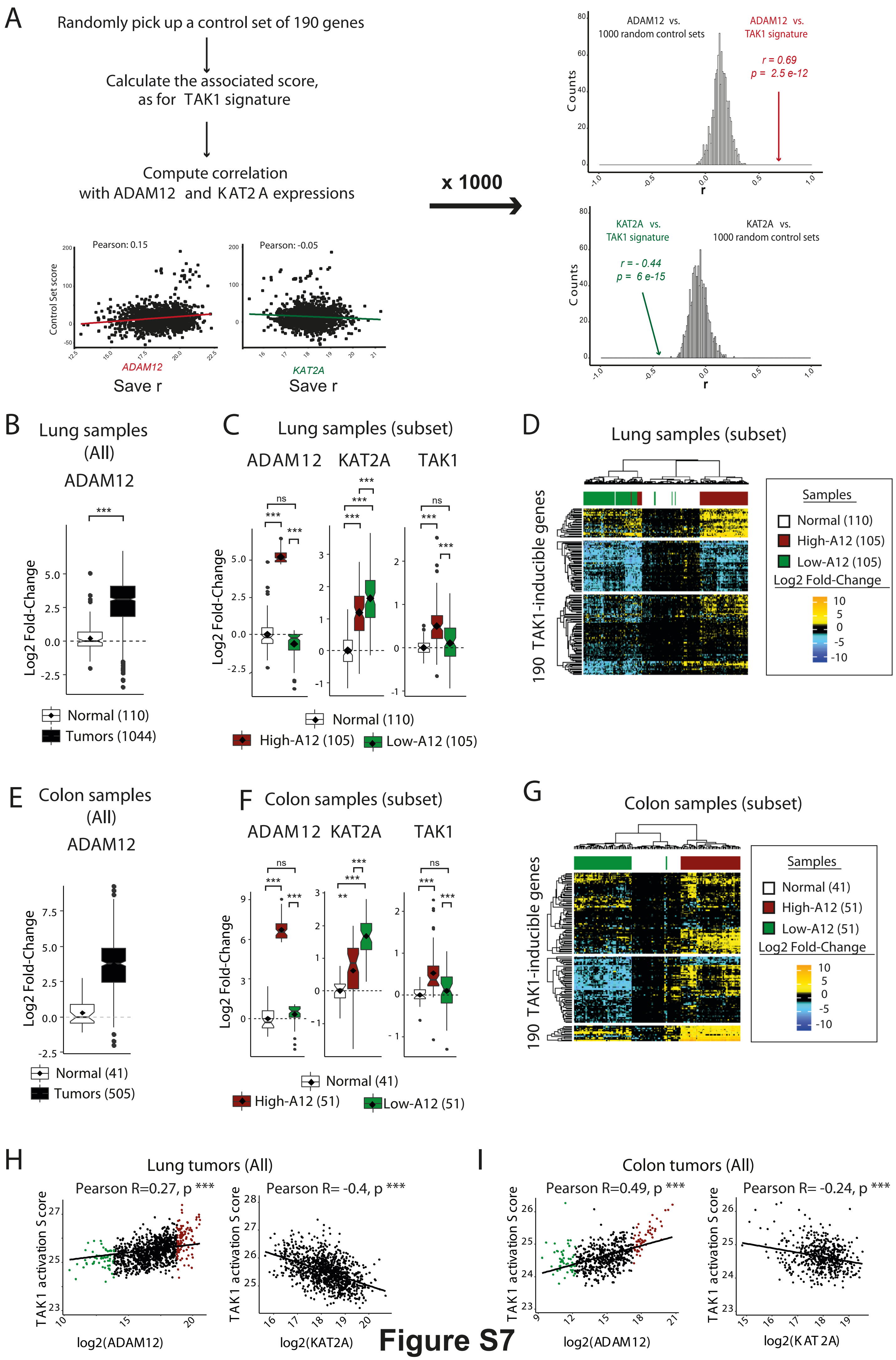

Figure S7

Naciri et al.

Table S1 : List of the kinases used in the screen

192 kinases genetically activated by addition of Myrystoylation sequence

All in pBabe vector (Hahn/Zhao; Addgene#1000000012)

AAK1	CKMT1A	HK1	MVK	PIP5K1B	RPS6KL1
ACVR1	CKMT2	HK2	NADK	PIP5K2A	RPSK6A3
ADCK4	CKS1B	HK3	NEK11	PIP5K3	SGK
ADCK5	CKS2	IHPK2	NEK3	PKM2	SNF1LK
ADPGK	CLK1	IKBKE	NEK6	PKN1	SPHK2
ADRBK1	CLK2	ILK	NME7	PKN2	SRPK2
ADRBK2	CLK3	ITK	NTRK3	PLAU	STK17B
AKT1	CMPK	ITPK1	NUAK2	PLK1	STK3
AKT3	CSNK1A1L	ITPKB	OXSR1	PLK2	STK32A
AMHR2	CSNK1E	LCK	PACSIN1	PLK3	STK32B
AURKA	CSNK1G1	LIMK1	PAK4	PLK4	STK32C
AXL	CSNK1G2	LIMK2	PAPSS1	PMVK	STK33
BLK	DAK	MAP2K5	PBK	PNKP	STK38L
BMX	DGKG	MAP2K6	PCK2	PRKAA1	STK4
BTK	DGUOK	MAP2K7	PCTK1	PRKACB	STK40
CALM2	DLG5	MAP3K14	PCTK2	PRKACG	SYK
CAMK1G	DYRK2	MAP3K6	PCTK3	PRKAG2	TAOK3
CAMK2B	DYRK4	MAP3K7	PDIK1L	PRKAR2A	TBK1
CAMK2D	EPHA4	MAP3K8	PDK1	PRKCD	TEC
CAMK4	FASTK	MAPK12	PDPK1	PRKCI	TESK1
CAMKK1	FGFR1	MAPK13	PDXK	PRKCZ	TIE1
CAMKV	FGR	MAPK14	PFKL	PRKRA	TK1
CDC2	FRK	MAPK6	PFKM	PTK2	TNK2
CDK2	GAK	MAPK7	PI4K2B	RET	TSSK1B
CDK4	GALK2	MAPKAP1	PIK3CB	RIOK1	TSSK6
CDK5	GCK	MAST1	PIK3CG	RIOK2	TTK
CDK7	GK	MATK	PIK3R3	RIOK3	TYK2
CDK9	GK2	MELK	PIK3R5	RPS6KA2	UCK2
CERK	GRK5	MKNK1	PIK4CA	RPS6KA5	ULK4
CHEK1	GRK6	MOBKL1A	PIK4CB	RPS6KA6	VRK2
CKB	HCK	MOBKL2A	PIM1	RPS6KB1	VRK3
CKM	HIPK1	MPP1	PIP5K1A	RPS6KB2	YES1

Table S2 : List of Nanostring probes

Probe number	symbol	Probe class	Accession #	Target Sequence
1	ADAM12	Tissue-restricted	NM_003474.4	GTACTGATGTCTCCCTCGCTCGAAATTACACGGTAATTCTGGGTCACTGTTACTACCATGGACATGTACGGGGATATTCTGATTCAGCAGTCAGTCTCAG
2	ADAM2	Tissue-restricted	NM_001464.3	AGGATTGTGCCCTTATTGGAGAAACATGCTGTGATATTGCCACATGTAGATTTAAAGCCGGTTCAAACTGTGCTGAAGGACCATGCTGCGAAAACTGTCT
3	ADCY10	Tissue-restricted	NM_001167749.1	CAAAACTGTCATTTTATGGGTCAGCTATAGACAGACATGGCGCTTCAGCTGTCTTCAGAATAATGTCACCCGGCCTCCTCTCCTGTCTTCTGCAGTCTTA
4	ALAS1	Tissue-restricted	NM_000688.4	GGGGATCGGGATGGAGTCATGCCAAAAATGGACATCATTTCTGGAACACTTGGCAAAGCCTTTGGTTGTGTTGGAGGGTACATCGCCAGCACGAGTTCTC
5	ASZ1	Tissue-restricted	NM_130768.2	GGTTCGGGTCCTTTTGGACAGAGGTGCTAATGCAAGCTTTGAGAAGGATAAGCAAAGTATTTTGATAACTGCATGTTCTGCTCATGGCTCAGAGGAACAG
6	ATAD2	Tissue-restricted	NM_014109.3	GAAAAACCTCGTCACCAGAGAAAGCCCAACATATTTTATAGTGGCCCAGCTTCTCCTGCAAGACCAAGATACCGATTATCTTCCGCAGGACCAAGAAGTC
7	BRDT	Tissue-restricted	NM_001726.3	ACAGATAGGATATTGTGTGCAAGACACAACCTCTGCCAATACTACCCTTGTTCATCAGACCACACCTTCACATGTAATGCCACCAAATCACCACCAATTA
8	C10ORF82	Tissue-restricted	NM_144661.2	AAGATGTATCTAGAGCCACTGTCCTCCGCAAAGTATGCAGAAGGCTAGAAGCGCAGAGTCTCCCAAGGAGGTGAACTTTAAGTGGGGCTTCCAAAACCTG
9	C9orf11	Tissue-restricted	NM_001161585.1	CCAGAGCTGGCCACGATGTCTTACTTTCATCCATCAGAAGGTGTTTCAGATACATCCTTTTCCAAGAGTGCAGAGAGCAGCACATTTTTGGGTACCACTT
10	CTAG1B	Tissue-restricted	NM_001327.2	GCGGGGCCAGGGGGCCGGAGAGCCGCCTGCTTGAGTTCTACCTCGCCATGCCTTTCGCGACACCCATGGAAGCAGAGCTGGCCCGCAGGAGCCTGGCCCA
11	CTCFL	Tissue-restricted	NM_001269042.1	TTAACACCCACACAGGAACCAGGCCCTACAAGTGTAACGACTGCAACATGGCATTTGTCACCAGTGGAGAACTCGTCCGACACAGGCGCTATAAACATAC
12	DAZL	Tissue-restricted	NM_001351.2	ACCTCCGGCTTATTCAGCTGTTAACTACCACTGTAATGAAGTTGATCCAGGAGCTGAAGTTGTGCCAAATGAATGTTCAGTTCATGAAGCTACTCCACCC
13	DDX4	Tissue-restricted	NM_024415.2	AGAAAGTAGTGATACTCAAGGACCAAAAGTGACCTACATACCCCCTCCTCCACCTGAGGATGAGGACTCCATCTTTGCACATTATCAGACAGGCATAAAC
14	DDX53	Tissue-restricted	NM_182699.2	ACAAACCGGAACAGGGAAAACATTGTCCTATCTAATGCCTGGGTTTATTCATCTTGATTCTCAACCAATATCTAGAGAGCAAAGGAATGGGCCTGGGATG
15	DPEP3	Tissue-restricted	NM_022357.3	CAGCGGATTGACAAGCTTTGGTGAGAAAGTAGTAGAGGAGTTGAACCGCCTGGGCATGATGATAGATTTGTCCTATGCATCGGACACCTTGATAAGAAGG
16	FMR1NB	Tissue-restricted	NM_152578.2	TCCTGGTATGTCTGCCCATTTATTGCCGCTCTCTTTTCTGGAGGAGCGAACCGGCCGATGATTTACAAAGGCAGGACAACAGAGTTGTAACGGGTTTGAA
17	HORMAD1	Tissue-restricted	NM_032132.3	ATTCAAATACACCAATAATGGACCACTCATGGACTTCATAAGTAAAAACCAAAGCAACGAATCTAGCATGTTGTCTACTGACACCAAGAAAGCAAGCATT
18	MAEL	Tissue-restricted	NM_001286377.1	CATCAGAAATCAGGCAAGATCTACAACTTCTCACTGTAGAGGACCTTGTAGTGGGGATCTACCAACAAAAATTTCTCAAGGAGCCCTCTAAGACTTGGAT
19	MAGEA12	Tissue-restricted	NM_001166386.1	CCCACTACCATCAACTATACTCTCTGGAGTCAATCCGATGAGGGCTCCAGCAACGAAGAACAGGAAGGGCCAAGCACCTTTCCTGACCTGGAGACGAGCT
20	MAGEA3	Tissue-restricted	NM_005362.3	ACTGTGCCCCTGAGGAGAAAATCTGGGAGGAGCTGAGTGTGTTAGAGGTGTTTGAGGGGAGGGAAGACAGTATCTTGGGGGATCCCAAGAAGCTGCTCAC
21	MAGEA4	Tissue-restricted	NM_001011548.1	TGATGGCCTGCTGGGTAATAATCAGATCTTTCCCAAGACAGGCCTTCTGATAATCGTCCTGGGCACAATTGCAATGGAGGGCGACAGCGCCTCTGAGGAG
22	MAGEB18	Tissue-restricted	NM_173699.3	AGAAATAAATCTCAGGATCCGGCAGGTGTCAAAGGTGCCCTCTTCATCAATTCAACTGCCTGCACTATTGTCTGTAGTCCCTGACAAGAGTAATTATGCC
23	MAGEB6	Tissue-restricted	NM_173523.2	TGGGAGTTCCTGGGTCTGTTGGGGATATATGATGGGATCCTGCATTCAATCTATGGGGATGCTCGGAAGATCATTACTGAAGATTTGGTGCAAGATAAGT
24	PIWIL1	Tissue-restricted	NM_001190971.1	TAACTCCAGAGCAAAGGCAGCGTGAAGTGGGACGACTCATTGATTACATTCATAAAAACGATAATGTTCAAAGGGAGCTTCGAGACTGGGGTTTGAGCTT
25	PLCZ1	Tissue-restricted	NM_033123.3	TGCATTCATGACATCTGACTACCCAGTGGTGCTCTCTTTAGAAAATCACTGCTCCACTGCCCAACAAGAAGTAATGGCAGACAATTTGCAGGCTACTTTT
26	PRAME	Tissue-restricted	NM_006115.3	AGGACCTGGTCTTTGATGAGTGTGGGATCACGGATGATCAGCTCCTTGCCCTCCTGCCTTCCCTGAGCCACTGCTCCCAGCTTACAACCTTAAGCTTCTA
27	RBM46	Tissue-restricted	NM_001277171.1	CTTATCCAGGCTATCCTTTGTCACCAACAATATCACTTGCTAATGGCAGCCATGTTGGACAGCGGCTATGTATCTCCAATCAGGCCTCCTTCTTCTGAAG
28	RFX4	Tissue-restricted	NM_213594.1	CTGCCAGCCAGCCTGCCTGAGGAGAAGGTTTCTACCTTTATTATGATGTACAGAACACACTGTCAGAGAATACTGGACACTGTAATAAGAGCCAACTTTG
29	RNF17	Tissue-restricted	NM_001184993.1	CCAGCACCAGAACAGATAGTGACATTATATGACGATGAACAGCATCCAGTTCATATGCCGTTGGTAGAAATGGGGCTTGCAGATAAAGATGAATAAGTGC
30	ROPN1	Tissue-restricted	NM_017578.2	TCCTGCCATCTACATGTGCCCAGAGTCCCCAAAATTGAGTGTCAAACAGAGGCTGGGTGACCTCCTGTCCAGGATTGCTCTCATCGTATGAATCGAAGTT
31	SPACA3	Tissue-restricted	NM_173847.3	TGCACTCAAGCCCTGTTTCTTCTCCTTCTGTGAGTGGACCACGGAGGCTGGTGAGCTGCCTGTCATCCCAAAGCTCAGCTCTGAGCCAGAGTGGTGGTGG
32	SPATA22	Tissue-restricted	NM_001170695.1	ACAGATTGGGCCTGGGAAGCTGTGAATCCAGAGTTGGCTCCTGTAATGAAAACAGTGGACACCGGGCAAATACCACATTCAGTTTCTCGTCCTCTGAGAA
33	SSX1	Tissue-restricted	NM_005635.2	CACTTTGTCACCAACTGCTGCCAACTCGCCACCACTGCTGCCGACCTCGCAACCACTGCTTTGTCTCTGAAGTGAGACTGCTCCTGGTGCCATGAACGGA
34	SSX2	Tissue-restricted	NM_003147.4	TACCTTAGAAAACAAGTATTGTCGGTTACCTCTGCATGGAACAGCATTACCCTCCTCTCTCCCCAGATGTGACTACTGAGGGCAGTTCTGAGTGTTTAAT
35	STK31	Tissue-restricted	NM_031414.2	GGATGTTGACACAGAAGCCAAGGTGATTGAGAGAGCAGCCACCTACCATAGAGCTTGGAGAGAAGCTGAAGGAGACTCAGGGTTACTGCCATTGATATTC
36	TKTL1	Tissue-restricted	NM_001145933.1	ACGTTTGAGAGGCTGTCAGGCAGCAGCGAAAGCTTGTTAGGATGTCCTGTGCTGCTTGTGATGAGAGCCTCCACACTGTACTGTTCAAGTCAATGTTAAT
37	TKTL2	Tissue-restricted	NM_032136.4	TGGTTTGGCTCTGGCTAAACTGGGCCGTGCAAATGAAAGAGTTATTGTTCTGAGTGGTGACACGATGAACTCCACCTTTTCTGAGATATTCAGGAAAGAA
38	TPTE	Tissue-restricted	NM_199259.2	TAGCTCCGCGCCCGAGAGAATGTTGGACCGACGACACAAGACCTCAGACTTGTGTTATTCTAGCAGCTGAACACACCCCAGGCTCTTCTGACCGGCAGTG
39	TSPYL6	Tissue-restricted	NM_001003937.2	TGGGTCACATCCTTCTAAGCGAAGCGAATGATGCTGTAGGTTACACTGTATTTACCTTTGTTTGGACTCGGTTTGTTCCCAGGACCTCTGGTCTGGCTCC
40	TUBA3C	Tissue-restricted	NM_006001.2	GGGCGTGCGCAGCTGCAGCGGCGGTTGAGGTCAAGTAGTAGCGTTGGGCTGCGGCAGCGGAGGAGCTCAACATGCGTGAGTGTATCTCTATCCACGTGGG
41	XAGE1	Tissue-restricted	NM_001097592.2	TGATCTGCAAGAGCTGCATCAGTCAAACACCGGGGATAAATCTGGATTTGGGTTCCGGCGTCAAGGTGAAGATAATACCTAAAGAGGAACACTGTAAAAT
42	XAGE3	Tissue-restricted	NM_133179.2	GGTGTGAGGGTCGCGTTCCTGCTGTCTGGACTTTTTCTGTCCCACTGAGACGCAGCTGTGTGAAATATGATTTGGCGAGGAAGATCAACATATAGGCCTA
43	DNMT1	Control	NM_001379.2	CAAAACCAATCTATGATGATGACCCATCTCTTGAAGGTGGTGTTAATGGCAAAAATCTTGGCCCCATAAATGAATGGTGGATCACTGGCTTTGATGGAGG
44	PPM1D	Control	NM_003620.2	CCACACTCTTGACCCTCAGAAGCACAAGTATATTATATTGGGGAGTGATGGACTTTGGAATATGATTCCACCACAAGATGCCATCTCAATGTGCCAGGAC
45	Tet1	Control	NM_030625.2	AATGCCAATCAGAAAGCCCATCCTTTGACCCAGCCCTCCTCTCCACCTAACCAGTGTGCTAACGTGATGGCAGGCGATGACCAAATACGGTTTCAGCAGG
46	Tet2	Control	NM_001127208.2	CTCATAATGTCCAAATGGGACTGGAGGAAGTACAGAATATAAATCGTAGAAATTCCCCTTATAGTCAGACCATGAAATCAAGTGCATGCAAAATACAGGT
47	Tet3	Control	NM_144993.1	ATCGCTTCGCAGCAGGTTCTCACAAAATAACTGGTGCTAGCTCAAGAAATCATCATCTGACCATCAGAAATCTTGACTAAAGGTGTTGCATGGATTTGGG
48	UHRF1	Control	NM_001048201.1	TGTGGAGGCTGTTGATGTTTCTGGTGTCAAGTTCTCAGAAGTTGCTGCCACCAACTCTTTAAGAAGGCGACAGGATCAGTCCTTCTCTCGGGTTCTGGCC
49	ZBTB33	Control	NM_006777.3	AGTCAGTACTTGGGTTGTGTAATCTGCTAGTCCAAGCGAACAGCAACCTCCTGCTACCCTCCCTCTATGAAAATAGCCATGCAGACAAGTCTCTCATCTG
50	ZBTB38	Control	NM_001080412.2	TTTGCCAGACACGGACCACATGGTTAAATTTGTTAATGGGCAAATGCTCTACAGTTGCGTTGTGTGCAAACGTAGTTATGTGACCTTATCTAGCCTCCGA
51	ZBTB4	Control	NM_020899.3	ACCTAATGACCCGCCTTGTTCTGAAGCTTTCTCTAAGCCCTTCCCAGTTGCTTCCTAGCACATTCCATTCTTTGTGGCCCAGGGCCTGGACCAGACCATT
52	MyrFlag	Normalizer	NA	ATGGGGTCTTCAAAATCTAAACCAAAGGACCCCAGCCAGCGCCGGCGCAGGATCCGAGGTTACCTTGACTACAAAGACGATGACGACAAGCAATTGAC
53	NeoR	Normalizer	NA	ACGCAGGTTCTCCGGCCGCTTGGGTGGAGAGGCTATTCGGCTATGACTGGGCACAACAGACAATCGGCTGCTCTGATGCCGCCGTGTTCCGGCTGTCAGC
54	PuroR	Normalizer	NA	GCCACGCGCCACACCGTCGATCCGGACCGCCACATCGAGCGGGTCACCGAGCTGCAAGAACTCTTCCTCACGCGCGTCGGGCTCGACATCGGCAAGGTGT
55	B2M	Normalizer	NM_004048.2	CGGGCATTCCTGAAGCTGACAGCATTCGGGCCGAGATGTCTCGCTCCGTGGCCTTAGCTGTGCTCGCGCTACTCTCTCTTTCTGGCCTGGAGGCTATCCA
56	PGK1	Normalizer	NM_000291.3	ATTGTCAAAGACCTAATGTCCAAAGCTGAGAAGAATGGTGTGAAGATTACCTTGCCTGTTGACTTTGTCACTGCTGACAAGTTTGATGAGAATGCCAAGA
57	TBP	Normalizer	NM_001172085.1	ACAGTGAATCTTGGTTGTAAACTTGACCTAAAGACCATTGCACTTCGTGCCCGAAACGCCGAATATAATCCCAAGCGGTTTGCTGCGGTAATCATGAGGA
58	TUBB2A	Normalizer	NM_001069.2	AGGACGAGGCTTAAAAACTTCTCAGATCAATCGTGCATCCTTAGTGAACTTCTGTTGTCCTCAAGCATGGTCTTTCTACTTGTAAACTATGGTGCTCAGT
59	NEG_A	internal controls	ERCC_00096.1	AACCGCCGCATACGGCCGATTGTCGCAGCCCGGGTCGATTATAACAACGGTGCAATCTCAGCTAAACCGACGCAGTTTTGCTCCTTGGATTCTGAGCCCG
60	NEG_B	internal controls	ERCC_00041.1	GCACTGGCATTGGTCGTTTCAGGAGGCCATACAGAACTGGTTTATATGAAGGAACATGGATCATTTGAAGTCATTGGGGAAACCCTTGATGATGCGGCAG
61	NEG_C	internal controls	ERCC_00019.1	GTACAGGCTGCTGGCTCATGTTTCCTTCTACGCTGCACTTGCGGGCATAGAGGTCGGTTGCGATCTATATTCGGAGATAACTATTCACCCAGCGCCACTC
62	NEG_D	internal controls	ERCC_00076.1	AGAGATCACGTGGACCAAAGCTGATTGATTACGGGACTGGCCGTAAGTGCTGCCCGCGAGTAGATCGTCTAGATCCGGCTAAAATTCCCTGCGGTGCCTT
63	NEG_E	internal controls	ERCC_00098.1	CCCAGATGACCTTCTCCCTCATAATCACTTAATCTGAGCGCAGGAGGCAGGCTGTATTAATTCCGGCCTCCAACCGGACCGTGGAACGACGCGACCAAGT
64	NEG_F	internal controls	ERCC_00126.1	GGGTCCTTACCGGCTGTAAGCTCACTACAATCCAGGTACAGAGTGCGTTAACCGGCCATTAGAGGGCCGCTACACCCGTCAGAATTTAAACGTATGGGCG
65	NEG_G	internal controls	ERCC_00144.1	ACCCGTATGAACTGTTGCCGGCTCGGAAATGTTAAGGCTCTGCGCACGCACTTTATCATTCGCAGCCTGTTCTGTCAGCGGGTCAGCCTAGGTTACGGTG
66	NEG_H	internal controls	ERCC_00154.1	TTGGTCCGAGGAGGCATATAGGAAACGATGGGCACGCGCTATTCAGACGTTATTTGGTATGGAGTAAGAGGCCGGAAACTGGGCTCGATTGATGGATACT
67	POS_A	internal controls	ERCC_00117.1	TCAGGCCTTGCCCTTACTAATGGCGCGTTGTAACGGGCCTTGAGGGAATGTCACTATTGAGGCACCCGTTCGACCCTCAGAGATATACCATTCCGCCTAT
68	POS_B	internal controls	ERCC_00112.1	ATGAAAGCGCTGCTACTATGATAAGAGTACACGTACAGGTCTCGCCCGATTGGATTATGGCGAGCTGCCGCATTGACGGACATACCTTTGAACGTAATCG
69	POS_C	internal controls	ERCC_00002.1	GCCGCTTTCGCTCGGGTCTGCGGGTTATAGCTTTTCAGTCTCGACGGGCTAGCACACATCTGGTTGACTAGGCGCATAGTCGCCATTCACAGATTTGCTC
70	POS_D	internal controls	ERCC_00092.1	TACCTGGCATTTGGGCACTTCTTGCGTTTAAGCGGGAAAGATCGCGAGGGCCCGCTATTTGCGATACTTCCCATGTCGGTGCCGTCGCCTCTATGTACTC
71	POS_E	internal controls	ERCC_00035.1	GGTTGAATTTGAGCGGATGGGCTCAACTGCGTCGTAACCGGTAGATACAGGGCATACGAGCCTCCCTATTTAACGGCATCATCCCGCGTAGTGCTGGTCA
72	POS_F	internal controls	ERCC_00034.1	ACGAAGCTGTTCGGCCGCACGCAAGTACCTCCCACTTAGAAAGCGAATAACCCAACGACCGTGTTCAACCCTGGCCGTCTCTCAACCAGGTATGCAATCA

Table S3 : List of tissue-restricted genes analyzed

	Gene	Tissue with highest expression	Cancer/Testis Antigen?	CpG island?	CpG island methylated in IMR90?
1	ADAM12	Placenta	No	Yes	Partially
2	ADAM2	Testis	Yes	No	
3	ADCY10	Testis	No	No	
4	ALAS1	Adrenal Gland/Liver	No	Yes	No
5	ASZ1	Testis	Yes	Yes	Yes
6	ATAD2	Testis/Bone marrow	Yes	Yes	No
7	BRDT	Testis	Yes	Yes	Yes
8	C10ORF82	Testis	No	Yes	Partially
9	C9orf11	Testis	No	No	
10	CTAG1B	Testis	Yes	No	
11	CTCFL	Testis	Yes	Yes	Yes
12	DAZL	Testis	No	Yes	Yes
13	DDX4	Testis	No	Yes	Yes
14	DDX53	Testis	Yes	No	
15	DPEP3	Testis	No	Yes	Yes
16	FMR1NB	Testis	Yes	Yes	Yes
17	HORMAD1	Testis	Yes	No	
18	MAEL	Testis	Yes	Yes	Yes
19	MAGEA12	Testis	Yes	No	
20	MAGEA3	Testis	Yes	Yes	No data
21	MAGEA4	Testis	Yes	No	
22	MAGEB18	Testis	Yes	No	
23	MAGEB6	Testis	Yes	No	
24	PIWIL1	Testis	Yes	Yes	Yes
25	PLCZ1	Testis	No	No	
26	PRAME	Testis	Yes	Yes	Yes
27	RBM46	Testis	Yes	Yes	Yes
28	RFX4	Testis	No	Yes	No
29	RNF17	Testis	No	No	
30	ROPN1	Testis	Yes	Yes	No data
31	SPACA3	Testis	Yes	No	
32	SPATA22	Testis	No	Yes	Yes
33	SSX1	Testis	Yes	No	
34	SSX2	Testis	Yes	No	
35	STK31	Testis	No	No	
36	TKTL1	Testis	No	Yes	Yes
37	TKTL2	Testis	No	Yes	Yes
38	TPTE	Testis	Yes	Yes	Yes
39	TSPYL6	Testis	No	Yes	Yes
40	TUBA3C	Testis	No	No	
41	XAGE1	Testis	Yes	No	
42	XAGE3	Placenta	Yes	No	

Table S4. List of 160 chromatin regulators targeted in the siRNA screen.

ARID1A	HDAC2	MBD6	SMARCA1
ASF1A	HDAC3	MECP2	SMARCA2
ASH1L	HDAC4	MEIS1	SMARCA4
ATRX	HDAC5	MPHOSPH8	SMARCA5
AZI2	HDAC6	PBRM1	SMARCB1
BMI1	HDAC7	PCGF1	SMARCC1
BPTF	HDAC8	PCGF2	SMARCC2
BRD1	HMGB1	PCGF3	SMARCD1
BRD2	HMGB3	PCGF5	SMARCD2
BRD3	HOXA5	PCGF6	SMCHD1
BRD4	INO80	PHC1	SUV39H1
BRD7	KAT2A	PHC2	SUV420H1
BRD8	KAT2B	PHF8	SUV420H2
CBX1	KAT6A	PPM1D	SUZ12
CBX3	KAT6B	PRDM2	TDG
CBX5	KDM1A	PRMT1	TET2
CBX6	KDM1B	PRMT5	TET3
CBX7	KDM2A	RBBP4	TRIM24
CDYL	KDM2B	RBBP7	TRIM28
CDYL2	KDM3A	RCOR1	TRIM33
CHD1	KDM3B	REST	UHRF1
CHD3	KDM4A	RING1	UHRF2
CHD4	KDM4C	RNF2	WTAP
CHD6	KDM4D	RXRA	YTHDC1
CHD8	KDM4E	SCAPER	YTHDF1
CTBP1	KDM5A	SCMH1	YTHDF2
CTBP2	KDM5B	SETD1A	YTHDF3
DICER1	KDM5C	SETD1B	ZBTB17
DLX5	KDM6B	SETD2	ZBTB33
DNMT1	KLF5	SETD7	ZBTB38
DNMT3A	KMT2A	SETD8	ZBTB4
DNMT3B	KMT2C	SETDB1	ZBTB40
EED	KMT2E	SETDB2	ZBTB44
EHMT2	LMNA	SIN3A	ZCCHC7
ELP3	LMNB2	SIRT1	ZHX1
EZH1	MBD1	SIRT2	ZHX2
EZH2	MBD2	SIRT3	ZNF114
HAT1	MBD3	SIRT6	ZNF217
HDAC1	MBD4	SIRT7	ZNF416
HDAC11	MBD5	SMAD4	ZNF695

Table S5. List of qRT-PCR primers.

Genes	Forward primers	Reverse primers
ADAM12	AGCTTATGGAACCAAGGAAGAG	CAGTTCTTTGCTTTCCCGTTG
TAK1	CGTCGGAAACCCTTTGATG	CGCTGGGAAGGATCTTTAGAC
KAT2A	CTGTGCTGTCACCTCGAATG	TCGGCGTAGGTGAGGAAGTA
SIRT6	CAGAGCTCCACGGGAACAT	ACGACTGTGTCTCGGACGTACT
IL6	GCCAGAGCTGTGCAGATGA	ATGTCCTGCAGCCACTGGT
SMAD7	CAGATTCCCAACTTCTTCTGGAG	GTAGAGCCTCCCCACTCTCG
CDKN2B	CGTTAAGTTTACGGCCAACG	GCATGCCCTTGTTCTCCTC
**Housekeeping genes**		
TBP	TGGCCCATAGTGTCTTTGC	TCCTAGAGCATCTCCAGCACA
PGK1	AGGATAAAGTCAGCCATGTGAG	CACAGGAACTAAAAGGCAGGA

Table S6: Sequence of siRNAs

Genes	Target sequences
**KAT2A pool**	AGGACAAAUUGGUGCCCGA<br>GUUCCUGGCAUUCGAGAGA<br>GCUACUACGUGACCCGGAA<br>ACUCAUGUCUUUGGGCGAA
**KAT2A individual**	CCAAGCAGGUCUAUUUCUACC
**SIRT6 pool**	CCAAGUGUAAGACGCAGUA<br>GUACAUCGCUGCAGAUCCG<br>CCAAAAGGUGAAGGCCAA<br>GAACUGGCGAGGCUGGUCU
**SIRT6 individual**	CCGGCUCUGCACCGUGGCUAAGG
**TAK1 pool**	GGACAUUGCUUCUACAAAU<br>GAGUGAAUCUGGACGUUUA<br>GGAAAGCGUUUAUUGUAGA<br>GCAAUGAGUUGGUGUUUAC
**TAK1 individual**	UGGCUUAUCUUACACUGGA

Table S7: List of genes in the TAK1 signature

1	ABCA1	CTGF	HBEGF	LRP4	P3H2	SLC35F2	UBASH3B
2	ADAM12	CTPS1	HECTD2	LRRC32	P4HA3	SLC38A5	UBL3
3	ADAM19	DACT1	HIVEP2	MAP3K4	PAG1	SLC46A3	UCK2
4	ADAMTS4	DCBLD1	HMCN1	MAP3K7CL	PDGFA	SMAD7	VAV3
5	AMIGO2	DGKI	HS3ST3B1	MEX3B	PGM2L1	SMURF1	VDR
6	ANGPTL4	DKK1	HTR1F	MFAP3L	PHLDA1	SNAI1	XYLT1
7	ANKRD44	DNAJB5	IER3	MIR181A2	PHLDB1	SNORD56B	ZBTB21
8	APCDD1L	DRP2	IER3	MIR181B1	PKIA	SOCS6	ZNF175
9	ARHGEF40	DUSP6	IER3	MIR181B2	PLEK2	SORBS2	ZNF281
10	ATP10A	E2F7	IFNE	MIR218-1	PLPP4	SPDL1	ZNF365
11	B4GALT1	EDN1	IGF2BP3	MIR221	PLXDC2	SPHK1	
12	BHLHE40	EGR2	IL11	MIR222	PMEPA1	SRPX2	
13	BMP2	ELN	IL6	MIR31	PNMA1	ST6GAL2	
14	BMPR1B	EPHB2	INHBA	MIR31HG	PNP	STARD13	
15	BMPR2	ERBB4	ITGA2	MIR503	PODXL	STK38L	
16	BPGM	ESM1	ITGB6	MIR503HG	PRDM1	SYT14	
17	BTBD11	ETV6	IVNS1ABP	MLXIP	PRICKLE2	TCF4	
18	C4orf26	FAM57A	JADE3	MSC	PTGS2	TGFBI	
19	C5orf46	FAP	JUNB	MURC	RASD2	TMC7	
20	CCIN	FMNL3	KCNJ15	MYOZ1	RASL11B	TMEM2	
21	CDC42SE1	FNDC1	KDR	NCF2	RELT	TMEM51	
22	CDH2	FOXP1	KLF10	NEDD9	RHOB	TNFAIP6	
23	CDK17	FRMD6	LAMC2	NFATC2	SCX	TNFAIP8L3	
24	CHRNA9	FSTL3	LEF1	NNMT	SCX	TNS1	
25	CHST11	FZD8	LHFPL2	NOX4	SEMA7A	TPM1	
26	CNIH3	GALNT10	LIMS1	NREP	SERPINE1	TRIB1	
27	COL4A1	GFPT2	LINC00312	NRP2	SH3PXD2A	TSHZ3	
28	COL4A2	GLIS3	LMCD1	NRP2	SKIL	TSPAN13	
29	COMP	GPAM	LOC79160	OLFM2	SLC19A2	TSPAN2	
30	CPN2	GPR183	LRIG1	OLFML2B	SLC25A32	UACA	
